# Multiscale Rheo-Optical and Scattering Signatures of Deformation-Induced Anisotropy in Aqueous Cellulose Nanocrystal Suspensions under Large-Amplitude Oscillatory Shear

**DOI:** 10.1021/acs.langmuir.6c03702

**Published:** 2026-09-25

**Authors:** Yoshifumi Yamagata, Ainaa Amirah Marzuki, Saki Otobe, Moe Araida, Taisuke Sato

**Affiliations:** † Business Unit Characterization, Anton Paar Japan K. K., 1st fl., Riverside Sumida, Tsutsumi-dori 1-19-9, Sumida-ku, Tokyo 131-0034, Japan; ‡ Research Institute for Science and Technology, Tokyo University of Science, 2641 Yamazaki,Noda-shi, Chiba 278-8510, Japan; § Anton Paar Malaysia Sdn Bhd, 6, Jalan Perpustakawan U1/62, Temasya Glenmarie, Seksyen U1, 40150, Shah Alam, Selangor Malaysia; ∥ Photonic Lattice, Inc., LABO CITY SENDAI, 6-6-3 Minami-Yoshinari, Aoba-Ku, Sendai-City, Miyagi 989-3204, Japan

## Abstract

Aqueous surface-charged cellulose nanocrystal (CNC) suspensions exhibit concentration-dependent viscosity growth, gelation, and deformation-induced anisotropy, but the concentration and strain-amplitude ranges over which these responses emerge are not necessarily the same. Here, low-shear rheology and linear viscoelasticity are combined with rheo-polarized imaging (Rheo-PI) and rheo-small-angle light scattering (Rheo-SALS) to compare mechanical, optical, and scattering signatures in the same CNC system. Analysis of the operational low-rate viscosity and the Winter–Chambon criterion separates an empirical viscosity-crossover concentration, *C*_cross_, from the sol–gel transition concentration, *C*_g_. Under fixed-frequency large-amplitude oscillatory shear (LAOS), nonphase-resolved but directly comparable Rheo-PI and Rheo-SALS readouts show that annularly averaged Rheo-PI retardation becomes detectable at lower concentrations and lower strain amplitudes than those required for the appearance of anisotropic X-shaped SALS patterns. The first optical transition approximately coincides with an apparent contraction of initially circular SALS patterns, whereas clearly directional X-shaped SALS patterns appear only at larger strain amplitudes. The X-shaped lobe geometry then evolves continuously with the strain amplitude, providing a later SALS-based signature of directional mesoscopic anisotropy under the imposed LAOS conditions. Together, these results provide a comparative multiscale map of deformation-induced anisotropy in CNC suspensions and distinguish viscosity crossover, gelation, local optical anisotropy, mechanical yielding, and anisotropic scattering signatures as related but noncoincident responses.

## Graphical abstract

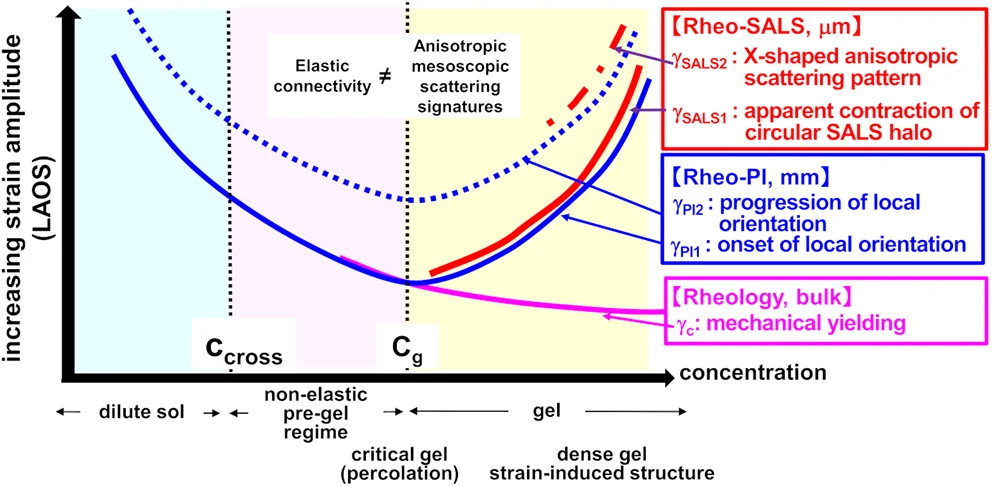

## Introduction

Gelation in aqueous suspensions of surface-charged rod-like colloids is governed by the interplay of concentration-dependent interparticle interactions, electrostatic stabilization, and deformation-induced orientation.^1–4^ In such systems, viscosity growth, the emergence of elastic connectivity, and anisotropic structural signatures do not necessarily appear under the same conditions or at the same length scale. For rod-like cellulose nanocrystal (CNC) suspensions, this distinction is particularly important because their large aspect ratio and orientational degrees of freedom can couple the rheological response to deformation-induced optical and scattering anisotropy.^5,6^ Clarifying when these observables emerge and whether they coincide or remain distinct is therefore essential for understanding the nonequilibrium organization of nanocellulose dispersions.

Cellulose nanocrystals (CNCs) and cellulose nanofibrils (CNFs) are naturally derived rod-like colloidal particles characterized by high rigidity and high aspect ratios, and they have been widely studied in soft matter physics and materials science.^7,8,11^ Aqueous suspensions of surface-charged nanocellulose are particularly useful model colloidal systems because electrostatic repulsion, concentration-dependent crowding, and externally imposed flow collectively determine their stability, gelation, and anisotropic organization.^11,12^ In such systems, the crystallinity, surface charge density, and aspect ratio of CNCs strongly influence their rheological properties and optical anisotropy.^9,12,13^

Previous studies have shown that the storage modulus and viscosity of CNC and CNF suspensions exhibit strong power-law dependencies on concentration,^10,12–14^ reflecting the development of elastic connectivity. Although single scaling relations can provide useful empirical descriptions over broad concentration ranges, the scaling of a single rheological observable does not by itself establish whether viscosity crossover, the emergence of a sample-spanning elastic network, and deformation-induced anisotropy correspond to the same physical transition. In particular, direct comparisons between concentration-dependent rheology and deformation-dependent structural responses within the same nanocellulose system remain limited.

In the Winter–Chambon framework, the sol–gel transition is identified as the concentration at which the storage and loss moduli exhibit the same power-law frequency dependence, G′∼G″ ∼ ω*^n^*, reflecting the emergence of a sample-spanning incipient elastic network in the linear regime.^15,16^ This criterion provides a rigorous mechanical definition of the sol–gel transition concentration, *C*_g_, but it does not by itself resolve deformation-induced orientation or anisotropic scattering under large-amplitude flow. In addition, quantities such as yield stress and yield strain are known to depend sensitively on the measurement protocol and observation method.^17^ Accordingly, optical and scattering observables obtained under LAOS should be interpreted as complementary structural readouts rather than as alternative definitions of the gel point.

To probe deformation-induced structural anisotropy more directly, rheo-optical techniques that combine rheological measurements with in situ structural characterization have attracted increasing attention. Rheo-small-angle light scattering (Rheo-SALS) enables direct visualization of anisotropic mesoscopic scattering patterns and heterogeneities during deformation and has been successfully applied to thixotropic clay gels, polymer–particle composites, and rod-like colloidal suspensions.^18,19^ More broadly, multiscale scattering approaches have revealed hierarchical structural development spanning particle-scale organization and larger-scale orientational correlations.^20,21^ More recently, we developed rheo-polarized imaging (Rheo-PI) based on two-dimensional polarization analysis, which allows simultaneous visualization of local optical anisotropy and orientation axes in transparent nanocellulose systems during rheological measurements.^22,23^ Applications of Rheo-PI to CNF suspensions have demonstrated its usefulness for tracking yielding-related structural evolution, but a direct comparison between Rheo-PI and Rheo-SALS responses within the same CNC system has not yet been systematically examined.

In this study, we use aqueous CNC suspensions as a model surface-charged rod-like colloidal system and compare concentration-dependent rheology with deformation-dependent rheo-optical and scattering responses under a common fixed-frequency LAOS protocol. Operational low-rate viscosity and linear viscoelasticity are used to distinguish an empirical viscosity-crossover concentration, *C*_cross_, from the sol–gel transition concentration, *C*_g_. Rheo-PI and Rheo-SALS are then used to follow nonphase-resolved but directly comparable optical and scattering responses as the strain amplitude is increased at selected concentrations. Fixed-frequency LAOS was chosen because it enables a controlled comparison of strain-amplitude-dependent mechanical, optical, and scattering responses from the linear to large-deformation regimes. It also provided more stable Rheo-PI and Rheo-SALS observations than continuous shear in the present optical geometry, where high-shear continuous-flow trials were prone to air entrainment and optical and scattering artifacts. Our objective is not to resolve intracycle structural evolution but rather to map when local optical anisotropy and anisotropic SALS signatures first become detectable relative to viscosity crossover, gelation, and mechanical yielding in the same CNC system.

By placing low-deformation rheology and fixed-frequency LAOS rheo-optical measurements in the same experimental framework, this study provides a comparative multiscale description of how viscosity crossover, gelation, local optical anisotropy, and anisotropic light scattering emerge in aqueous CNC suspensions. This comparison allows us to distinguish responses associated with concentration-dependent elastic connectivity from those associated with deformation-induced orientational organization and thereby to clarify how different observables become detectable under experimentally accessible conditions in a surface-charged rod-like colloidal gel.

## Experimental Methods

### Sample Preparation

The cellulose nanocrystals (CNCs) used in this study were purchased from Cellulose Lab (Canada) as a freeze-dried powder of a highly sulfated CNC (CNC-HS-FD) in the sodium salt form (sulfur content: approximately 1 wt %, CAS No. 9004-34-6). The CNC powder was used as received without any additional chemical treatment. Aqueous CNC suspensions were prepared by dispersing a prescribed amount of the CNC powder in purified water. The mixture was homogenized using a planetary centrifugal mixer (SK-300SII, Shashin Kagaku Co., Ltd., Kyoto, Japan) at 2000 rpm for 5 min, followed by a degassing step at 2000 rpm for 5 min. This mixing–degassing procedure was repeated twice to obtain homogeneous CNC suspensions. No dedicated quiescent liquid-crystalline phase characterization, such as polarized optical microscopy of resting samples, static SALS, or long-time phase-separation analysis, was performed in the present study. During visual handling, transient iridescent or birefringent colors were observed when transparent lower-concentration suspensions were shaken, indicating sensitivity to orientation-induced optical anisotropy; however, this observation was not used to assign an equilibrium liquid-crystalline phase state.

### Viscosity and Frequency Sweep Measurements

Viscosity measurements and dynamic viscoelastic frequency sweep tests of the CNC suspensions were performed using a stress-controlled rheometer (MCR 302e, Anton Paar GmbH, Graz, Austria). A cone–plate geometry with a cone angle of 1° was employed, and cone diameters of either 50 mm or 25 mm were selected depending on the viscosity of the sample. For the viscosity measurements, flow curves were obtained by continuous shear-rate sweep experiments in which the shear rate was exponentially increased from 0.01 to 1000 s^–1^; thus, the reported viscosities were not acquired as independently equilibrated steady-state values at each shear rate. For samples exhibiting a clear low-shear plateau, η_low_ was taken from that plateau region. For samples without a well-defined low-shear plateau, the viscosity at 0.01 s^–1^ was used as a practical low-shear estimate of η_low_ for comparative analysis. Therefore, the concentration dependence of η_low_ discussed later should be interpreted as an operational result obtained under the present sweep protocol. Frequency sweep measurements were conducted at a fixed strain amplitude of 1%, within the linear viscoelastic regime while sweeping the angular frequency from 0.01 to 100 rad s^–1^. No reverse shear-rate sweep or stepped steady-state hysteresis protocol was performed in the present study; therefore, thixotropic hysteresis was not quantified from these measurements. All measurements were carried out at 20 °C, and a solvent trap was used to prevent water evaporation during the experiments.

### Rheo-Polarized Imaging (Rheo-PI, Rheo-Iris) Measurements under Fixed-Frequency LAOS

A schematic illustration of the rheo-polarized imaging (Rheo-PI, Rheo-Iris) setup is shown in Figure 1(a).^23^ A high-speed polarization camera capable of real-time measurement of optical retardation and orientation-axis distributions (CRYSTA PI-5WP, Photonic Lattice, Inc., Japan) was installed beneath the rheometer. The rheometer head was equipped with a light source consisting of a ring-shaped white LED combined with a circular polarizing film. A band-pass filter centered at 543 nm was mounted in front of the polarization sensor so that only light around 543 nm was used for the quantitative retardation measurement. The polarization camera simultaneously records four polarization-resolved intensities at each local position by using four adjacent polarization pixels. From these intensities, the full Stokes parameters are calculated, and the phase retardation and principal axis orientation are determined by comparison with the corresponding Stokes parameters measured without the sample. The optical retardation, *R*, is then obtained from the phase retardation, δ, using the measurement wavelength according to *R* = δλPI/(2π), where δ is the phase retardation in radians and λ_PI_ = 543 nm. Here, *R* represents the optical path difference arising from birefringence along the optical path and is used as a quantitative measure of local optical anisotropy in the transparent CNC suspensions. Further technical details of the Rheo-PI/Rheo-Iris method and analysis procedure are provided in ref 23. The transmitted light was captured by the polarization camera located below the sample. Here, the 1-, 2-, and 3-directions denote the flow, velocity-gradient, and vorticity directions, respectively. Because the transmitted light was collected along the velocity-gradient direction, the Rheo-PI maps correspond to optical anisotropy projected onto the flow–vorticity plane, i.e., the 1–3 plane. Measurements were performed using a quartz upper plate with a diameter of 43 mm and a glass lower plate with a fixed gap of 0.5 mm. Because the local strain amplitude in the parallel-plate geometry varies with the radial position, the observation radius must be specified. For the Anton Paar rheometer operated with the parallel-plate geometry, the nominal shear rate and strain amplitude are evaluated at the effective measuring radius, reff = 2*R*/3. In the present geometry, the upper plate diameter was 43 mm, corresponding to *R* = 21.5 mm and reff = 14.3 mm. The Rheo-PI retardation, ΔPI, was quantified in a fixed annular region centered at approximately *r* = 15 mm from the fixture center. This position was selected to match the Rheo-SALS laser-beam position and is close to the effective measuring radius used for the nominal strain convention. Large-amplitude oscillatory shear (LAOS) tests were conducted at a fixed angular frequency of 10 rad s^–1^ while sweeping the strain amplitude over the range γ = 0.001–100.

**Figure 1:**
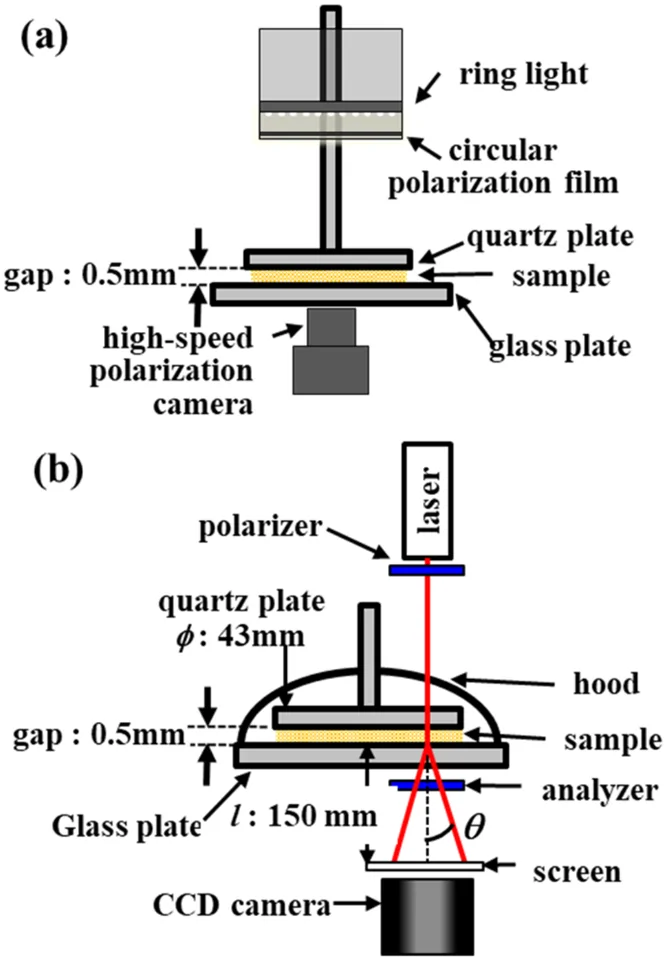
Schematic illustrations of the experimental setups for (a) rheo-polarized imaging (Rheo-PI, Rheo-Iris) and (b) rheo-small-angle light scattering (Rheo-SALS). In both configurations, the optical axis is approximately along the velocity-gradient direction, and the observed image/scattering plane corresponds to the flow–vorticity plane, i.e., the 1–3 plane.

Dynamic viscoelastic properties were simultaneously measured with acquisition of the polarized images. Polarized images were recorded continuously during the strain-amplitude sweep without synchronization to the oscillation phase. Accordingly, the Rheo-PI images and Δ_PI_ values presented in this study are treated as nonphase-resolved, acquisition-window-averaged, condition-dependent optical readouts under the imposed LAOS conditions. They are therefore not interpreted as intracycle-resolved structural quantities. In addition, because dedicated fixed-amplitude time-resolved measurements were not performed, they are not used to establish rigorously whether a time-independent periodically driven state was reached at each strain amplitude. All experiments were carried out at 20 °C, and a solvent evaporation protection hood was used throughout the measurements.

### Rheo-Small-Angle Light Scattering (Rheo-SALS) Measurements under LAOS

A schematic illustration of the Rheo-SALS setup is shown in Figure 1(b). A laser module (Anton Paar GmbH, Graz, Austria) was mounted above the rheometer, and the laser beam was introduced parallel to the rotational axis, passing through the sample. Under this geometry, the incident beam propagated approximately along the velocity-gradient direction. Therefore, the recorded SALS patterns probe scattering anisotropy in the plane perpendicular to the beam, corresponding to the flow–vorticity plane, i.e., the 1–3 plane. The transmitted and scattered light was projected onto a screen, and the scattering patterns were recorded using a CCD camera. The laser wavelength, λ_SALS_, was 658 nm, and the sample-to-screen distance (shadow distance) was set to 150 mm. The SALS laser beam had a diameter of approximately 1 mm and was positioned with its center at approximately *r* = 15 mm from the fixture center. This position was chosen to match the annular region used for the Rheo-PI Δ_PI_ analysis. Therefore, Rheo-PI and Rheo-SALS sampled equivalent local strain positions in the parallel-plate geometry. Polarization measurements were performed in the crossed configuration (HV), with the polarizer and analyzer orthogonally oriented. To obtain the optimal scattering intensity, the exposure time and camera gain were adjusted depending on the CNC concentration (Table S1). The same geometry and fixed-frequency LAOS protocol, including the dimensionless strain-amplitude range γ = 0.001–100, as those used in the Rheo-PI measurements were applied for the Rheo-SALS experiments. Scattering images were recorded continuously during the strain-amplitude sweep without synchronization to the oscillation phase. Accordingly, the SALS patterns presented in this study are treated as nonphase-resolved, acquisition-window-averaged, condition-dependent scattering readouts under the imposed LAOS conditions. They are therefore not interpreted as intracycle-resolved scattering structures. In addition, because dedicated fixed-amplitude time-resolved measurements were not performed, they are not used to establish rigorously whether a time-independent periodically driven state was reached at each strain amplitude. All measurements were conducted at 20 °C along with solvent evaporation protection.

The accessible scattering vector is determined by the optical geometry of the Rheo-SALS setup. Using the laser wavelength λ_SALS_ = 658 nm, the sample-to-screen distance *l* = 150 mm, and the detector pixel density *p* = 84.854 pixels cm^–1^, the scattering vector corresponding to a detector position *x* was calculated as

1 $$q=\frac{4\pi }{\lambda_{\mathrm{SALS}}}\;\sin\left(\frac{\theta }{2}\right)=\frac{4000\pi }{\lambda_{\mathrm{SALS}}}\;\sin\left\{\frac{1}{2}a\;\tan\left(\frac{\left(x/p\right)}{l}\right)\right\}$$

In the annular-intensity analysis described below, the range *q* = 0.28–0.58 μm^–1^ was used, corresponding to characteristic length scales 2π/*q* ≈ 22.4–10.8 μm. This *q* range was selected for consistent comparison of anisotropic SALS features across concentrations and strain amplitudes under the present optical geometry.

## Results and Discussion

### Viscosity Crossover and Elastic Connectivity

In this section, we show that aqueous cellulose nanocrystal (CNC) suspensions exhibit distinct concentration regimes associated with a crossover in the concentration dependence of the operational low-rate viscosity and the subsequent formation of an infinitely connected elastic network capable of supporting stress. In the present aqueous suspension of surface-charged rod-like colloids, the concentration dependence of η_low_ should be interpreted as reflecting not only geometrical crowding but also electrostatic and hydrodynamic interactions under the present measurement protocol. Because viscosity also increases in the dilute regime, an increase in viscosity alone is not taken as evidence for a distinct physical transition. Instead, the change in the slope of the operational low-rate viscosity is treated here as an empirical viscosity crossover between the low- and high-concentration regimes. This viscosity crossover must be clearly distinguished from the emergence of elasticity. The concentration-dependent measurements discussed in this section should therefore be interpreted as characterizing how viscous crossover and elastic connectivity evolve with concentration prior to large deformation. They are conceptually distinct from the Rheo-PI and Rheo-SALS results discussed below, which probe strain-amplitude-dependent structural responses at fixed concentration under LAOS.

Figure 2(a) shows the viscosity curves of CNC suspensions at various concentrations. At a CNC concentration of 0.75 wt %, the viscosity remained nearly constant over the entire measured shear-rate range, exhibiting a behavior close to Newtonian flow. When the CNC concentration was increased to 1.5–4 wt %, a shear-thinning behavior was observed at high shear rates, whereas a well-defined low-shear plateau appeared at low shear rates. At CNC concentrations of 5 wt % and higher, the viscosity exhibited pronounced shear thinning over the entire measured shear-rate range, and a clear low-shear Newtonian plateau could no longer be identified. For samples exhibiting a clear low-shear plateau, η_low_ approximates the zero-shear viscosity η_0_. For samples without a well-defined low-shear plateau, η_low_ was estimated operationally as the apparent viscosity at 0.01 s^–1^ for comparative analysis. This value should not be interpreted as a fitted or independently determined zero-shear viscosity, η_0_. We did not extract η_0_ by fitting the high-concentration flow curves because such an estimate would require extrapolation below the measured shear-rate range and would be strongly dependent on the assumed model and fitting range. Based on these results, the concentration dependence of the operational low-rate viscosity η_low_ is summarized in Figure 2(b).

**Figure 2:**
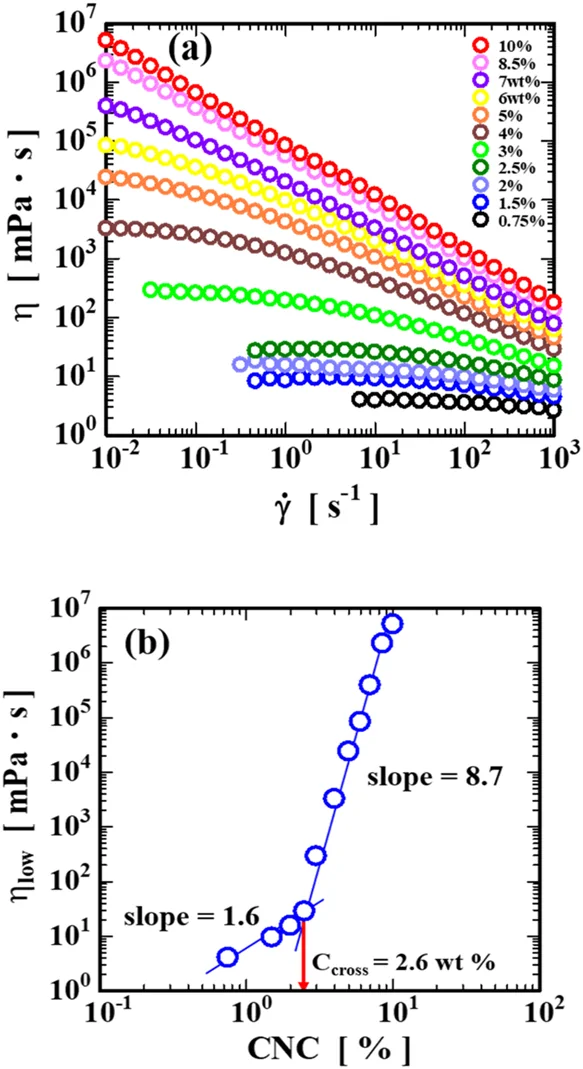
Concentration-dependent flow behavior of aqueous CNC suspensions. (a) Viscosity as a function of shear rate for aqueous CNC suspensions at various concentrations (0.75–10 wt %), showing the transition from nearly Newtonian behavior to pronounced shear thinning. (b) Concentration dependence of the operational low-rate viscosity, ηlow, showing two empirical power-law regimes and their intersection, which defines the empirical viscosity-crossover concentration, *C*_cross_ = 2.6 wt %.

As shown in Figure 2(b), the operational low-rate viscosity η_low_ exhibits two distinct power-law regimes as a function of the CNC concentration, with a clear crossover between the low- and high-concentration regions. In this study, the concentration at which these two fitted regimes intersect is denoted *C*_cross_ and is used only as an empirical viscosity-crossover concentration. The resulting crossover concentration was *C*_cross_ ≈ 2.6 wt %. Because this value was obtained from a limited number of concentration points and depends on the selected fitting ranges, it is treated as an approximate operational crossover concentration rather than as a sharply defined critical concentration. It should not be identified with the canonical overlap concentration c* in the Doi–Edwards sense. In the low-concentration regime, the operational low-rate viscosity follows

2 $$\eta_{\mathrm{low}}\propto c^{1.6}$$

whereas in the experimentally accessible high-concentration regime, a much steeper apparent dependence is observed

3 $$\eta_{\mathrm{low}}\propto c^{8.7}$$

The two exponents obtained here, 1.6 and 8.7, are treated as empirical slopes describing the two concentration regimes observed for the present aqueous CNC suspensions. They should not be interpreted as universal scaling exponents for ideal rigid rods. In particular, the present system differs from an idealized neutral-rod picture because the suspensions are surface-charged and aqueous, interparticle interactions are influenced by electrostatic repulsion and hydration, and η_low_ at higher concentrations was estimated operationally from continuous shear-rate sweep data rather than from independently equilibrated zero-shear measurements. Accordingly, the role of these fitted slopes in the present study is not to establish a universal concentration scaling law but to identify a practical crossover concentration, *C*_cross_, for comparison with the independently determined sol–gel transition concentration, *C*_g_.

To further examine whether the rapid viscosity increase could be described by a divergence-like concentration dependence, we also attempted a Vogel–Fulcher–Tammann (VFT)-type fit to the apparent low-rate viscosity data using

4 $$\eta_{\mathrm{app}}\left(C\right)=\eta_{\mathrm{pre}}\;\exp\left(\frac{A}{C_{0}-C}\right)$$

where *C*_0_ is the apparent divergence concentration. The fit was applied to the positive apparent-viscosity data at 0.01 s^–1^ in the concentration range of 3–10 wt % because the lower-concentration data at this shear rate were strongly affected by low-torque measurement uncertainty. Although the VFT-type expression qualitatively reproduced the steep increase in apparent viscosity, the fitted *C*_0_ was highly sensitive to the imposed constraint and fitting condition, as summarized in Table S2. For example, *C*_0_ moved to the imposed upper bounds of 12, 15, 20, and 50 wt %, while an upper bound of *C*_0_ ≤ 100 wt % gave *C*_0_ = 92.9 wt %, and the fit without an upper bound on *C*_0_ gave an even larger value of approximately 190 wt %. Because these fitted divergence concentrations lie far outside the measured concentration range and are not robustly determined, the VFT-derived *C*_0_ was not used as a material parameter or as a direct counterpart of *C*_g_. We therefore retain *C*_cross_ only as an operational viscosity-crossover concentration under the present measurement protocol.

### Mechanical Yielding and Transition to Plasto-Viscoelastic Response

In this section, we examine the linear viscoelastic and LAOS responses of CNC suspensions in order to define concentration- and strain-based mechanical reference quantities for subsequent comparison with the rheo-optical and scattering results. We examine the stress–strain behavior and dynamic viscoelastic responses of CNC suspensions under large-amplitude oscillatory shear (LAOS) and demonstrate that the mechanical yield characterizes a transition from a solid-like elastic response to a plasto-viscoelastic response. Importantly, we show that this mechanically defined yield does not necessarily coincide with the emergence of orientation or structural anisotropy, which will be discussed in subsequent sections.

We first considered the linear viscoelastic properties obtained from frequency sweep measurements. Figure 3 shows the angular frequency (ω) dependence of the storage modulus G′ and loss modulus G″ for selected CNC concentrations. To improve the visibility, the moduli for different concentrations are vertically shifted by a constant factor *A*. For CNC concentrations of 5 wt % and lower, data at angular frequencies below 0.1 rad s^–1^ are not shown because the measured torque fell below the instrument resolution limit in this range. The reproducibility of the frequency sweep measurements and the absence of significant time-dependent structural evolution at low frequency were independently confirmed; details are provided in the Supporting Information (Figure S1). At the CNC concentration of 3 wt %, G′ remains smaller than G″ over the entire frequency range, and both moduli exhibit a pronounced frequency dependence, indicating that the system is in a fluid-like sol state. In contrast, at CNC concentrations of 8.5 wt % and higher, G′ exceeds G″ and both moduli show only a weak frequency dependence, demonstrating the formation of a gel state dominated by elasticity. Therefore, η_0_ was also not estimated from the low-frequency limit of G″/ω because the high-concentration samples did not exhibit a clear terminal viscoelastic regime within the accessible frequency range. Such an estimate would require G″ ∝ ω and a terminal flow response at low frequency, which were not observed for the gel-like samples studied here.

**Figure 3:**
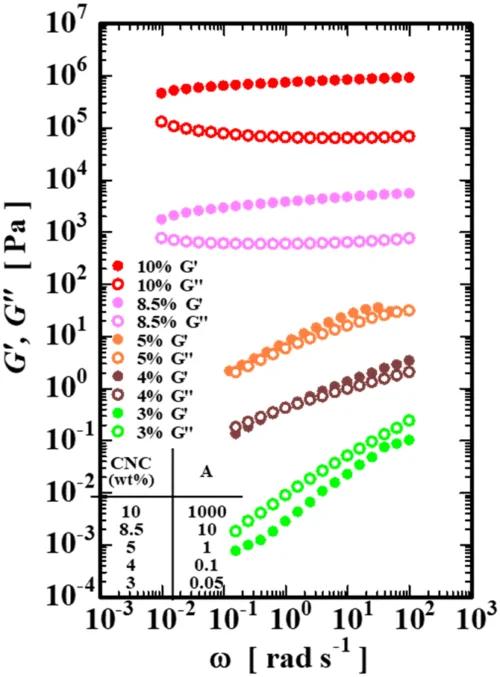
Angular frequency (ω) dependence of the storage modulus G′ (filled symbols) and loss modulus G″ (open symbols) for selected CNC concentrations. The intermediate concentration range showing approximately comparable power-law behavior is used to locate the approximate sol–gel transition region and to define *C*_g_ ≈ 4.5 wt % as a concentration-based mechanical reference.

At intermediate concentrations around 4–5 wt %, G′ and G″ show approximately comparable frequency dependences over the experimentally accessible frequency range

$$G^{\prime }\left(\omega \right)∼G^{\prime \prime }\left(\omega \right)∼\omega^{n},\;n\approx 0.5$$ 5

and appear as nearly parallel straight lines on a log–log plot. This behavior is consistent with the vicinity of a Winter–Chambon-type sol–gel transition.^15,16^ However, the common power-law behavior is not sharply resolved at a single concentration in the present dataset. In particular, additional concentration points around 4–5 wt % would be required for a more precise determination of the gel concentration. Therefore, in the present study, the sol–gel transition is regarded as being located approximately within the 4–5 wt % concentration range. For subsequent comparisons, *C*_g_ ≈ 4.5 wt % is used as an approximate concentration-based mechanical reference, corresponding to the midpoint of this transition interval, rather than as a precisely determined critical concentration. It should be emphasized that the sol–gel transition defined by the Winter–Chambon method reflects the emergence of an infinitely connected elastic network in the linear response regime and does not necessarily imply the appearance of orientation or structural anisotropy under large deformation. In the present study, *C*_g_ is therefore used as a concentration-based mechanical reference point for comparison with the deformation-dependent optical and scattering signatures discussed below. Neither the overlap concentration c* nor the isotropic–nematic boundary was independently determined for the present CNC batch because such determination would require additional information on the effective particle aspect-ratio distribution, electrostatic effective diameter, ionic strength, and quiescent phase coexistence. Therefore, *C*_cross_ and *C*_g_ are not identified with either c* or the isotropic–nematic transition. In the present paper, *C*_cross_ ≈ 2.6 wt % and *C*_g_ ≈ 4.5 wt % are used only as operational rheological and mechanical reference concentrations, respectively, rather than as equilibrium phase-boundary concentrations.

We next examined the dynamic viscoelastic response under LAOS conditions. Figure 4 shows the strain-amplitude (γ) dependence of G′ and G″ for three representative concentrations; i.e., a sol state (3 wt %), a concentration near the sol–gel transition (5 wt %), and a gel state (10 wt %). At all the concentrations, both moduli remain nearly constant in the low-strain region (≈10^–3^–10^–1)^, defining a clear linear viscoelastic (LVE) regime. In this regime, the internal microstructure undergoes minimal deformation, and the response primarily reflects the properties of the equilibrium elastic network. For the 10 wt % sample, G″ exhibits a distinct maximum at γ ≈ 0.13. Such an overshoot in G″ has been widely reported for soft materials and gels, and is commonly interpreted as a transient enhancement of energy dissipation accompanying the transition from the linear viscoelastic regime to a large-strain regime where the mechanical yield or structural breakdown begins.^24,25^ This behavior reflects a crossover from solid-like viscoelastic dissipation to flow-dominated (plastic) dissipation associated with structural yield.

**Figure 4:**
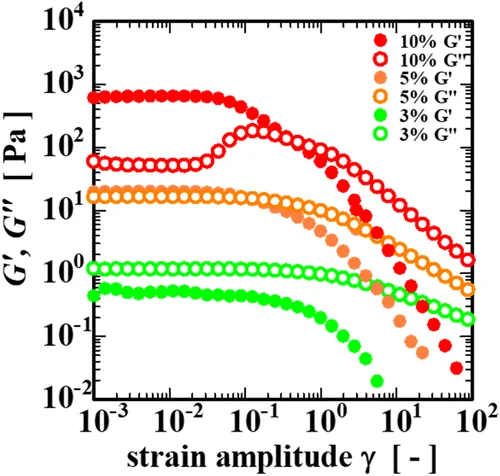
Strain-amplitude dependence of the storage modulus G′ (filled symbols) and loss modulus G″ (open symbols) for CNC suspensions at concentrations of 3, 5, and 10 wt % under fixed-frequency large-amplitude oscillatory shear (LAOS).

The plateau values of the moduli in the linear viscoelastic (LVE) regime are defined as the apparent plateau moduli G′_N_ and G″_N_. Figure 5 shows these plateau moduli plotted as a function of the excess concentration relative to the sol–gel transition (*c*–*C*_g_). In the gel regime above *C*_g_, both moduli increase following clear power-law dependences on (*c*–*C*_g_)­

6 $${G^{\prime }}_{N}\propto {\left(c-C_{g}\right)}^{1.54}$$

7 $${G^{\prime \prime }}_{N}\propto {\left(c-C_{g}\right)}^{0.56}$$

**Figure 5:**
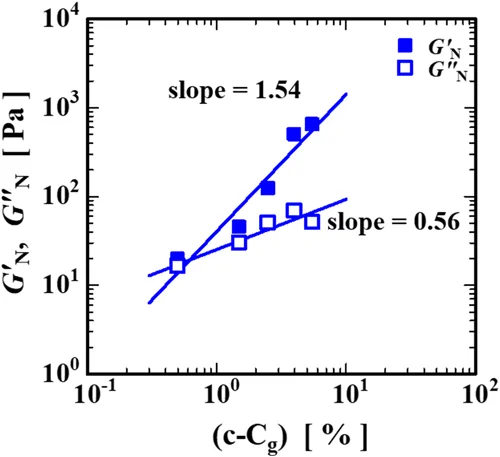
Scaling of the apparent plateau moduli G′_N_ and G″_N_ as a function of excess concentration above the sol–gel transition (*c*–*C*_g_), illustrating concentration-dependent strengthening of the gel network.

These scaling relations are obtained from data in the experimentally accessible concentration range above *C*_g_ (5–10 wt %) and provide a quantitative description of the growth of network stiffness (G′_N_) and energy dissipation (G″_N_) in the gel regime.

The exponent of 1.54 for G′_N_ is comparable to values often reported for colloidal and physical gels, indicating that the development of elastic connectivity is dominant in the present gel regime.^1,2^ In contrast, the smaller exponent for G″_N_ suggests that energy dissipation is primarily governed by localized relaxation processes and reversible bond rearrangements. Thus, the different concentration dependences of G′_N_ and G″_N_ indicate that elastic stiffness and viscous dissipation evolve differently in the present gel regime.^1^ It is important to distinguish these concentration-dependent rheological trends from the strain-amplitude-dependent structural responses discussed in the following sections. The concentration scaling of G′_N_ and G″_N_ reflects how elastic connectivity strengthens with increasing concentration under low-deformation/linear conditions and is not interpreted here as evidence of flow-induced orientation. By contrast, the Rheo-PI and Rheo-SALS results presented below probe deformation-induced optical and mesoscopic anisotropy at fixed concentration under LAOS. Possible concentration-induced orientational ordering at rest is not the quantity addressed by the present analysis of G′_N_ and G″_N_. Accordingly, G′_N_, G″_N_, and *C*_g_ are treated here as low-deformation mechanical reference quantities, whereas the Rheo-PI and Rheo-SALS observables are treated as deformation-dependent structural readouts under the fixed-frequency LAOS protocol.

Finally, stress–strain (σ–γ) curves were constructed from the LAOS data (Figure S2), and the mechanical critical strain γ_c_ was defined as the point at which the slope deviates from unity in the low-strain regime. For γ < γ_c_, the material exhibits an elastic, solid-like response, whereas for γ > γ_c_, irreversible deformation occurs beyond the yield point and a plasto-viscoelastic response dominated by a coexistence of the elastic, viscous, and plastic contributions becomes prevalent.^25,26^ As shown in Figure 6, γ_c_ decreases with increasing CNC concentration, indicating that the transition from elastic to plasto-viscoelastic behavior occurs at progressively lower strains in more concentrated systems. Within the present fixed-frequency LAOS protocol, γ_c_ is therefore used as an operational mechanical yielding threshold for comparison with γ_PI1_, γ_PI2_, γ_SALS1_, and γ_SALS2_, rather than as a protocol-independent material constant. This mechanically defined critical strain, γ_c_, will be discussed in detail in the following sections together with the critical strains at which pronounced changes in the anisotropy are observed in the rheo-optical measurements, namely, γ_PI1_, γ_PI2_, γ_SALS1_, and γ_SALS2_.

**Figure 6:**
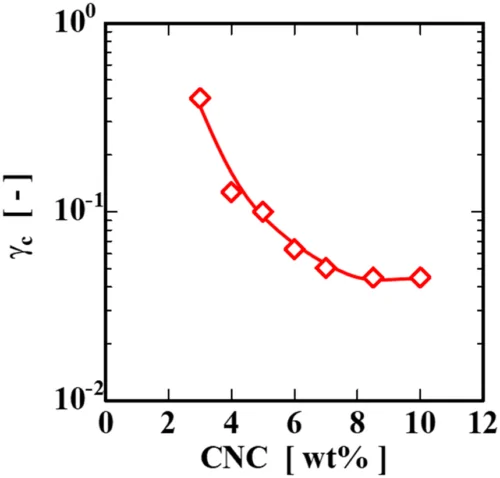
Concentration dependence of the mechanical critical strain, γ_c_, determined from stress–strain curves under fixed-frequency LAOS.

Possible wall slip or edge fracture at large strain amplitudes should also be considered when interpreting the LAOS stress response and the large-γ optical observations. In the present study, no independent quantitative wall-slip test, such as a rough-plate comparison, gap-dependence test, or velocimetric measurement, was performed. However, visual inspection after the measurements did not reveal macroscopic sample expulsion from the fixture edge or obvious edge fracture. In addition, the stress–strain curves did not show an abrupt collapse of stress with increasing strain amplitude. These observations suggest that pronounced wall slip or edge fracture did not dominate the reported transition strains, although minor local slip at the largest strain amplitudes cannot be completely excluded.

Because the mechanical-transition strain can depend on the chosen criterion, alternative mechanical criteria were also examined. In addition to γ_c_, defined from the deviation of the stress–strain curve from the low-strain linear response, we evaluated the strain amplitude at which G″ reaches a maximum, the strain amplitude at which the elastic stress amplitude G′γ reaches a maximum, and the G′ = G″ crossover strain, when the crossover was observed within the measured strain-amplitude range. Here, G′γ was used as the elastic stress amplitude. The resulting values are summarized in Table S3.

These alternative criteria gave different absolute strain amplitudes. The strain at maximum G″ ranged from 0.00113 to 0.127, depending on CNC concentration, whereas the strain at maximum G′γ occurred at larger strain amplitudes of approximately 1.0–1.4. The G′ = G″ crossover was observed only for CNC concentrations of 5 wt % and higher, with the crossover strain increasing from 0.100 to 0.400 as the CNC concentration increased. Thus, the mechanical-transition strain is criterion-dependent. In the present study, γ_c_ is retained as an operational marker for the onset of nonlinear mechanical response, whereas the alternative criteria are used to assess the robustness of the comparison with the Rheo-PI and Rheo-SALS transition strains.

At higher CNC concentrations, the stress–strain curves also show features consistent with a two-stage nonlinear mechanical response. The first characteristic strain corresponds to γ_c_, which represents the onset of nonlinear mechanical response or initial structural rearrangement. In addition, for the higher-concentration samples, especially 6–10 wt %, a broad second bend is observed around γ ≈ 1. This later feature is consistent with the strain range where the elastic stress amplitude G′γ reaches its maximum. Because the position of this later bend is broad and remains close to γ ≈ 1 over the high-concentration range, it is not treated here as a unique concentration-dependent second yield strain. Instead, the maximum of G′γ is used as an operational marker of the later large-deformation mechanical response. This later mechanical marker occurs at larger strain amplitudes than γ_c_ and is closer to the second Rheo-PI and Rheo-SALS transition strains, but it does not provide a universal one-to-one correspondence with the optical or scattering transitions.

### Early Emergence of Optical Anisotropy Revealed by Rheo-PI

In this section, we turn from concentration-dependent rheological characterization to deformation-dependent optical readouts at fixed concentrations. Using Rheo-PI under the fixed-frequency LAOS protocol described above, we focus on when local optical anisotropy first becomes detectable relative to *C*_cross_, *C*_g_, and γ_c_, rather than attempting to resolve intracycle structural evolution. Optical anisotropy arising from local orientation of cellulose nanocrystals (CNCs) becomes detectable at lower concentrations and lower strain amplitudes than those associated with elastic connectivity and mechanical yield. Accordingly, any concentration dependence discussed in this section refers to differences in the deformation-induced optical response among samples of different concentrations, not to concentration-induced orientational ordering at rest. Because highly sulfated CNC suspensions can exhibit lyotropic chiral-nematic, biphasic, or kinetically arrested orientational states, the optical retardation measured here should not be interpreted exclusively as flow-induced alignment from a strictly isotropic initial state. In the present study, the quiescent liquid-crystalline phase state was not independently classified. Therefore, Δ_PI_ is treated as an operational optical anisotropy readout under the imposed LAOS condition, which may include contributions from deformation-induced alignment, preexisting local orientational order, and shear-modified orientational domains.

On the basis of the rheological results presented in the preceding sections, the concentration marking the viscosity crossover and the concentration at which an infinitely connected elastic network emerges can be clearly distinguished by operational low-rate viscosity and linear viscoelastic measurements. Furthermore, the concentration scaling of the plateau moduli in the linear viscoelastic regime provides a quantitative measure of the network stiffness after gelation. However, these rheological measurements are inherently based on spatially averaged mechanical responses and cannot directly capture the orientation, anisotropic structures, or heterogeneous rearrangements that develop within the network under a large deformation. This limitation is particularly critical for CNC systems composed of rod-like particles, in which shear deformation is known to induce particle orientation and microstructural evolution that are closely coupled to nonlinear viscoelastic responses and yield behavior. Rheo-PI is therefore used here as a complementary optical readout under imposed LAOS conditions to compare the onset and progression of local anisotropy to the mechanical reference quantities defined in the preceding sections.

Figure 7 shows representative polarized imaging maps of CNC suspensions under LAOS. These maps are presented as obtained under the imposed LAOS condition at each strain amplitude. Because the image acquisition was not synchronized to the oscillation phase, these maps should be interpreted as nonphase-resolved, acquisition-window-averaged optical maps acquired during the strain-amplitude sweep. They therefore provide condition-dependent structural readouts under the imposed LAOS conditions, rather than intracycle-resolved images. In each image, a central circular region with a radius of 12.7 mm, corresponding to the nontransparent part of the measurement fixture, is masked in black. Within the observation area, optical retardation is color-coded from dark blue (0 nm) to brown (270 nm), and the same retardation scale is applied for all the CNC concentrations. For the CNC suspension at 5 wt %, the evolution of polarized images under LAOS is also provided as a video in Figure S3. For CNC suspensions with concentrations of 6 wt % and higher, the true optical retardation exceeds the principal measurable range of the present optical configuration (approximately 270 nm) even at low strain amplitude. Because the measured retardation is periodic with respect to the true optical retardation, the apparent retardation then folds back, leading to phase-inverted, artifact-like images in the color-coded maps. This apparent inversion does not indicate a real decrease in optical anisotropy but is a measurement artifact arising from the periodic retardation response. Therefore, in Figure 7, only representative data for CNC concentrations of 5 wt % and lower, which are not affected by this folding, are shown. As can be seen from the figure, even for the low-concentration system at 2 wt %, which is below both *C*_cross_ and *C*_g_ and therefore outside the gel regime, the optical retardation slightly increases with increasing strain amplitude, indicating that flow-induced local orientation can develop under LAOS even in a liquid-like suspension. In contrast, this trend is much more pronounced for the 5 wt % system, whose concentration exceeds the sol–gel transition concentration *C*_g_.

**Figure 7:**
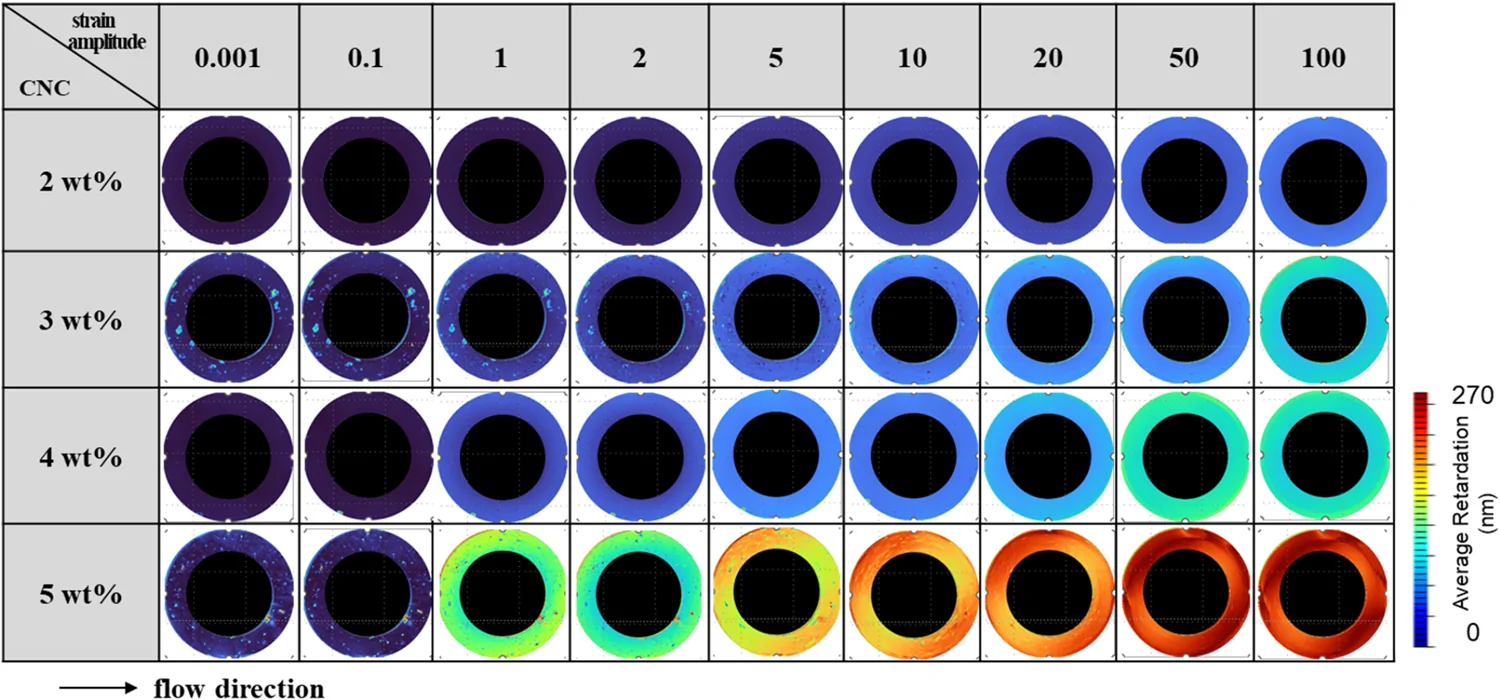
Representative rheo-polarized imaging (Rheo-PI) maps of aqueous CNC suspensions under fixed-frequency large-amplitude oscillatory shear (LAOS). The central opaque region (radius 12.7 mm) is masked in black, and the optical retardation is color-coded from 0 to 270 nm. Only data for CNC concentrations of 5 wt % and lower are shown; for higher concentrations, the retardation exceeded the principal measurable range of the present optical configuration and was reconstructed by phase unwrapping (see Figures 8 and S4).

In a parallel-plate geometry, the local strain amplitude varies with the radial position. However, this radial dependence is not always visually obvious in the representative retardation maps of Figure 7 because the optical response reflects not only the local strain amplitude but also the nonlinear orientational response. Therefore, rather than relying on direct radial comparison in Figure 7, we quantitatively evaluated the strain-amplitude dependence of the average retardation, Δ_PI_, within a fixed annular region centered at approximately *r* = 15 mm from the fixture center. This annular region was selected to match the Rheo-SALS laser-beam position. Here, Δ_PI_ denotes the average optical retardation calculated within this fixed annular region and is used as a representative scalar measure of the local optical anisotropy at that radial position. Thus, Δ_PI_ is not a whole-image average over the full field of view, but a quantity extracted from a defined observation region for consistent comparison under the parallel-plate geometry. The observation radius of *r* ≈ 15 mm is close to the effective measuring radius, reff = 2*R*/3 ≈ 14.3 mm for the 43 mm upper plate, used for the nominal strain convention of the Anton Paar parallel-plate geometry. Therefore, the Rheo-PI and Rheo-SALS transition strains are compared at equivalent local strain positions, and the strain amplitudes plotted in Figures 8 and 9 are retained as the imposed nominal strain amplitudes for consistency with the rheological control variable. Because the Rheo-PI images were not synchronized to a specific phase of the oscillatory cycle, Δ_PI_ should be interpreted as a nonphase-resolved, acquisition-window-averaged retardation value under each imposed LAOS condition rather than as an instantaneous intracycle retardation. The results for CNC suspensions with concentrations of 5 wt % and lower are shown in Figure 8(a) while those for concentrations of 6 wt % and higher are presented in Figure 8(b). For the latter systems, the measured optical retardation was found to exceed the detection limit (270 nm) with increasing strain amplitude, resulting in apparent retardation inversion. This apparent inversion is a consequence of the periodic measurement response and does not indicate an actual decrease in birefringence. Therefore, for CNC concentrations of 6 wt % and higher, the true optical retardation was obtained by applying a phase-unwrapping procedure to calculate Δ_PI_. Details of the phase-unwrapping procedure are provided in the Supporting Information (Figure S4). To avoid overlap between datasets obtained at different concentrations, the Δ_PI_ values shown in Figure 8(a) and (b) are vertically shifted by a constant factor *A*. As can be seen from Figure 8(a) and (b), the average retardation exhibits two distinct transition points: an initial transition characterized by a rapid increase in Δ_PI_ beyond a critical strain amplitude (indicated by the red arrows), followed by a second transition at a higher strain amplitude where the increase becomes more gradual (indicated by the black arrows). The corresponding strain amplitudes are defined as the critical strains γ_PI1_ and γ_PI2_, respectively, and their concentration dependence is summarized in Figure 9. Both γ_PI1_ and γ_PI2_ decrease with increasing concentration up to the sol–gel transition concentration *C*_g_ but exhibit an increasing trend again in the high-concentration gel regime above *C*_g_. This behavior can be interpreted under the fixed LAOS angular frequency used here (ω = 10 rad s^–1^). Up to the sol–gel transition concentration *C*_g_, the decrease of γ_PI1_ and γ_PI2_ with increasing concentration indicates that, under the fixed LAOS angular frequency used here, smaller strain amplitudes are sufficient to induce detectable optical anisotropy in more concentrated suspensions. This trend is consistent with the competition between the imposed deformation-rate amplitude and the orientational relaxation of the CNCs: because the angular frequency was fixed, increasing strain amplitude increases the deformation-rate amplitude, whereas increasing concentration is expected to reduce the orientational relaxation rate. Accordingly, lower strain amplitudes can induce measurable anisotropy at higher concentrations under the present conditions. However, because the LAOS angular frequency was fixed at ω = 10 rad s^–1^, the strain amplitude γ_0_ and the shear-rate amplitude γ̇_0_ = ω γ_0_ were not independently varied. Therefore, the present dataset cannot determine whether the observed Rheo-PI and Rheo-SALS transition amplitudes are controlled primarily by strain amplitude, shear-rate amplitude, or a combination of both. In this paper, γ_PI1_, γ_PI2_, γ_SALS1_, and γ_SALS2_ are therefore retained as operational transition amplitudes under the fixed-frequency LAOS protocol. In the gel regime above *C*_g_, however, the trend reverses, indicating that additional network constraints hinder further development of macroscopic orientation and anisotropy even after the transition to a plasto-viscoelastic state. In addition, Figure 10 provides representative orientation-axis maps extracted from the Rheo-PI measurements. Unlike Figure 7, which shows the magnitude of the optical retardation, Figure 10 visualizes the local principal optical axis determined from the Rheo-PI analysis. In these maps, the local orientation angle is indicated by the color/angle legend and displayed as short line segments. Under the present acquisition condition, the local orientation field tends to become more aligned with the flow direction as the optical retardation increases. This observation describes only the representative structural response under the imposed LAOS condition at each strain amplitude; it does not establish whether the oriented state persists after shear cessation or whether a time-independent steady state is reached at a fixed strain amplitude because dedicated cessation and fixed-amplitude time-resolved measurements were not performed in the present study. Accordingly, the increase in optical anisotropy detected by Rheo-PI is interpreted only as reflecting stronger local alignment and greater spatial coherence of the orientation field under the imposed LAOS condition. In this sense, the Rheo-PI results define a concentration- and strain-dependent optical reference that can be directly compared with the SALS signatures discussed in the next section.

**Figure 8:**
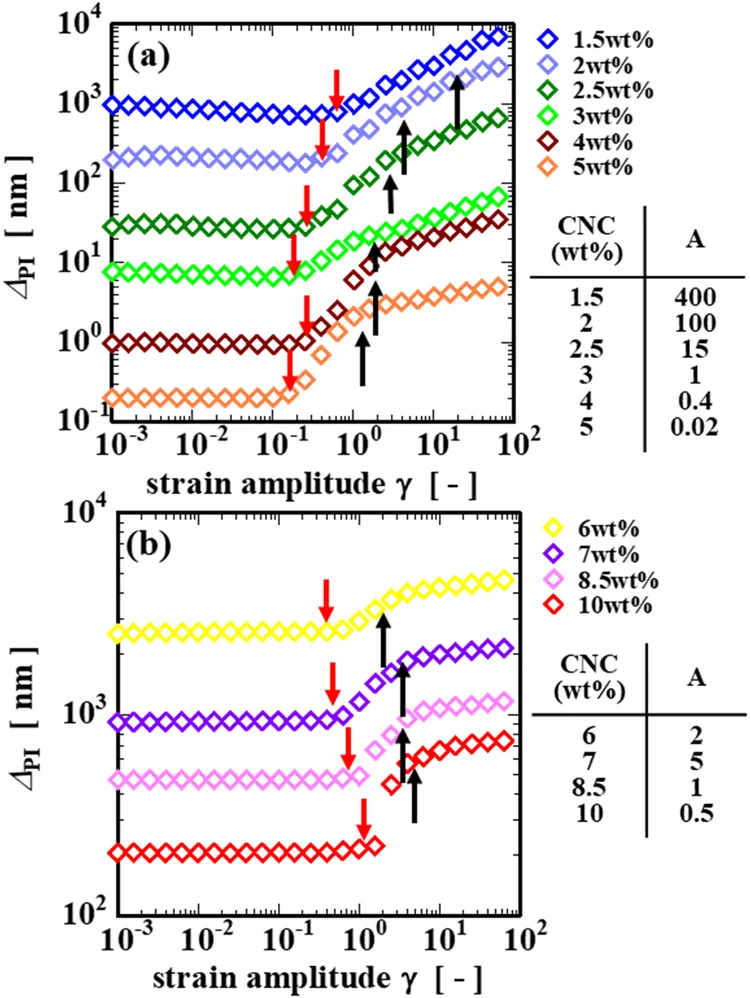
Strain-amplitude dependence of the average optical retardation, Δ_PI_, evaluated within a fixed annular region at a radial position of approximately 15 mm from the sample center under fixed-frequency LAOS. Results for CNC suspensions with concentrations of 5 wt % and lower are shown in panel (a), whereas those for concentrations of 6 wt % and higher are shown in panel (b). For concentrations of 6 wt % and higher, ΔPI was obtained after phase unwrapping of the measured retardation (see Figure S4). Red and black arrows indicate the first and second optical transitions, respectively.

**Figure 9:**
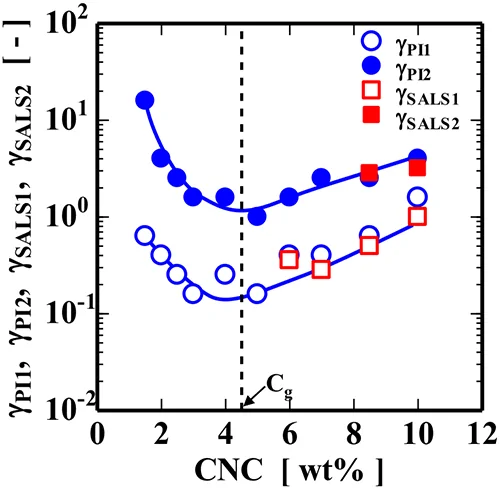
Concentration dependence of the critical strains determined by Rheo-PI and Rheo-SALS under fixed-frequency LAOS. γ_PI1_ and γ_PI2_ are obtained from Rheo-PI measurements, whereas γ_SALS1_ and γ_SALS2_ are defined from the apparent contraction of initially circular SALS patterns and the emergence of X-shaped SALS patterns, respectively.

**Figure 10:**
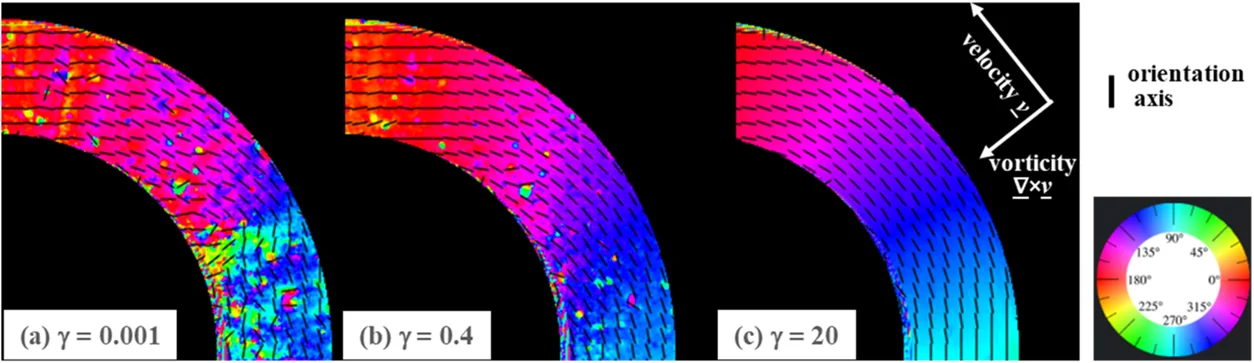
Representative orientation-axis maps extracted from the Rheo-PI measurements under fixed-frequency LAOS at selected strain amplitudes. Unlike Figure 7, which shows the magnitude of the optical retardation, this figure visualizes the local principal optical axis determined from the Rheo-PI analysis. The local orientation angle is indicated by the color/angle legend and displayed as short line segments.

### Delayed Development of X-Shaped Anisotropic SALS Patterns during Large Deformation

In this section, we use rheo-small-angle light scattering (Rheo-SALS) to examine how anisotropic mesoscopic scattering signatures develop under LAOS. Compared with the onset of local optical anisotropy detected by Rheo-PI, pronounced directional mesoscopic correlations become apparent only at larger strain amplitudes. Thus, under the present fixed-frequency LAOS protocol, Rheo-SALS is used as a complementary scattering readout to identify when larger-scale directional correlations become detectable relative to the optical transitions defined in the preceding section.

As shown by the polarized imaging results in the previous section, even at CNC concentrations below *C*_cross_, the optical retardation increases with increasing strain amplitude, and the representative orientation-axis maps qualitatively indicate increased flow-directional alignment under the imposed LAOS condition. To evaluate how such a local orientation appears on the mesoscopic scale, we next performed Rheo-SALS measurements. Figure 11 shows the evolution of the H_V_ scattering patterns under large-amplitude oscillatory shear (LAOS) for CNC suspensions with concentrations of 6 wt % and higher. In all the images, the flow direction is from left to right. To ensure sufficient visibility of the scattering patterns, the detector sensitivity and intensity scale were optimized for each CNC concentration. Due to imperfect alignment, the laser-beam position was slightly shifted downward relative to the beam stopper; however, a similar offset was observed for all the scattering patterns, allowing a reliable relative comparison. Therefore, this misalignment does not affect the discussion of strain-induced development of anisotropic mesoscopic scattering signatures. For the 10 wt % CNC suspension, the simultaneous evolution of the viscoelastic moduli (G′ and G″) and the scattering patterns under LAOS is also provided as a video (Figure S5). As in the Rheo-PI measurements, the SALS patterns were recorded continuously during the strain-amplitude sweep without synchronization to the oscillation phase and are therefore interpreted here as nonphase-resolved, acquisition-window-averaged, condition-dependent scattering readouts under the imposed LAOS conditions.

**Figure 11:**
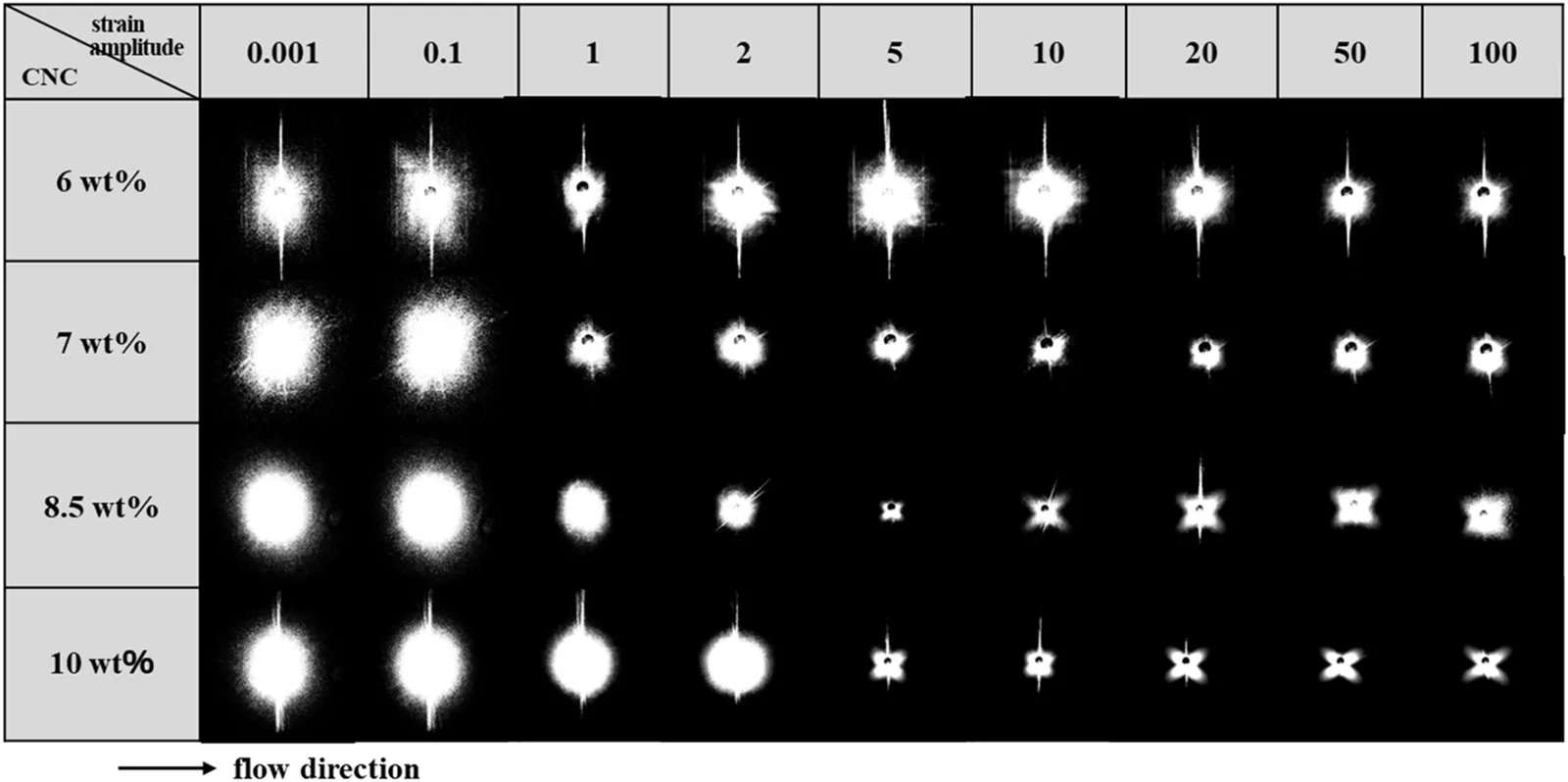
Evolution of Rheo-SALS HV scattering patterns under fixed-frequency large-amplitude oscillatory shear (LAOS) for aqueous CNC suspensions at concentrations of 6 wt % and higher. The scattering patterns evolve from nearly circular or weakly elliptical shapes at low-to-intermediate-strain amplitudes to clearly resolved anisotropic X-shaped patterns at higher strain amplitudes.

In the following discussion, we focus on the shape and anisotropy of the scattering patterns rather than on their absolute intensity. At a strain amplitude of γ = 0.001, for the CNC concentrations of 7 wt % and lower, the scattering patterns are slightly distorted but remain nearly circular. This nearly isotropic shape persists in the low-strain region. In contrast, for the higher-concentration samples, especially 8.5 and 10 wt %, weak ellipticity or weak pre-X anisotropy can be recognized at intermediate-strain amplitudes before the clearly resolved X-shaped pattern appears. Therefore, the intermediate-strain SALS response should not be interpreted as strictly isotropic for all high-concentration samples.

In these systems, the apparent radius of the circular or weakly distorted HV scattering halo begins to decrease when the strain amplitude reaches a certain value, which is defined here as the critical strain amplitude γ_SALS1_. In the revised interpretation, γ_SALS1_ is treated as an operational marker for the onset of this apparent radial contraction of the HV scattering pattern under the present optical geometry. We do not use γ_SALS1_ alone as direct evidence for a quantitatively determined increase in correlation length. Demonstrating correlation-length growth would require radial I­(*q*) cuts showing a reproducible shift of the dominant scattering feature toward lower *q* and separating this effect from changes in depolarized intensity or average optical retardation. For the 8.5 and 10 wt % samples, the weak ellipticity observed before the X-shaped pattern suggests that some orientational bias may already be present at this stage. However, this weak pre-X anisotropy is gradual and does not provide a sharply defined transition strain over the full concentration range.

Upon further increasing the strain amplitude, directional correlations between the CNC orientational domains become more pronounced, and the circular or weakly elliptical scattering pattern transforms into a clearly identifiable anisotropic X-shaped pattern. The critical strain amplitude associated with this transition is defined as γ_SALS2_. Thus, γ_SALS2_ is retained here as an operational marker for the onset of the clearly resolved four-lobed X-shaped SALS pattern, rather than for the first possible weak anisotropic distortion. The strain amplitude γ_SALS2_ is located in a strain-amplitude range comparable to the critical strain amplitude γ_PI2_, at which the increase in Δ_PI_ changes from a rapid rise to a more gradual growth (Figure 9). Taken together, these results indicate that Rheo-PI and Rheo-SALS report different but complementary stages of deformation-induced anisotropy: local optical anisotropy and weak mesoscopic anisotropy can appear first, whereas clearly resolved X-shaped SALS patterns emerge only after larger-scale directional correlations have developed. The appearance of the four-lobed anisotropic SALS pattern is particularly informative for interpreting the mesoscopic response. In the present paper, the term “X-shaped” refers specifically to the four-lobed HV scattering pattern observed under the crossed-polarization configuration used in the present Rheo-SALS measurements. We do not treat “cross-shaped” and “X-shaped” as distinct structural states. Rather, both expressions refer to the same type of anisotropic four-lobed scattering signature, and the apparent shape depends only on the angular orientation of the lobes relative to the flow direction. What is physically important here is therefore not the terminology itself, but the transition from a nearly circular pattern to a four-lobed anisotropic pattern, indicating the development of directional mesoscopic correlations. Because the present measurements were performed in the *H*_V_ configuration, our discussion is restricted to the depolarized *H*_V_ scattering signatures observed under this optical geometry, and we do not claim that the same pattern would appear identically in *H*_H_ or unpolarized SALS measurements.^27^

For CNC concentrations of 8.5 wt % and higher, X-shaped scattering patterns are clearly observed. The four lobes initially appear near ±45° relative to the flow direction, corresponding to the extensional and compressional axes, and their angular positions change with the increasing strain amplitude. To quantify this change, annular-intensity analysis was performed. Vertical streaks were observed near ϕ = 90° and 270° in some SALS images. A separate blank optical check performed without a sample showed that similar streaks appeared or disappeared when the lower glass plate was rotated, confirming that these streak components originated from the optical path or fixture, rather than from vorticity-aligned structural scattering of the CNC suspensions. They were most likely caused by residual dust or contamination on the glass plate or other optical components. Therefore, to avoid including these nonsample contributions in the azimuthal analysis, angular regions near ϕ = 90° and 270° were masked using ImageJ. Annular-intensity distributions were calculated in the scattering vector range *q* = 0.28–0.58 μm^–1^ (corresponding to characteristic length scales of 2π/*q* ≈ 22.4–10.8 μm), in which four-lobe peaks were clearly identified (Figure S6).

The peak azimuthal angles near 45°, 135°, 225°, and 315° are defined as μ_45_, μ_135_, μ_225_, and μ_315_, respectively, and their strain-amplitude dependences are shown in Figure 12(a). As the strain amplitude increases, μ_45_ and μ_225_ slightly decrease, whereas μ_135_ and μ_315_ show a slight increase.

**Figure 12:**
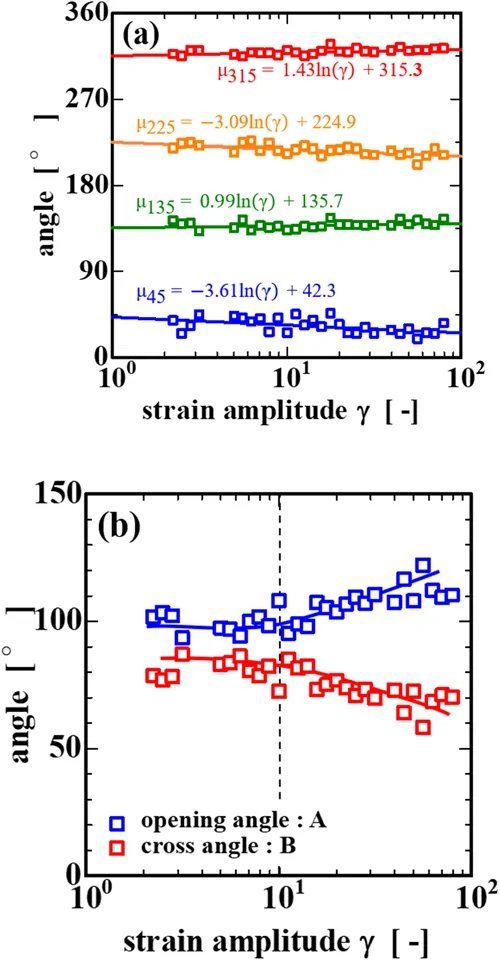
Quantitative analysis of the X-shaped Rheo-SALS patterns. (a) Strain-amplitude dependence of the azimuthal peak angles μ_45_, μ_135_, μ_225_, and μ_315_ extracted from the annular-intensity analysis of the X-shaped scattering patterns. (b) Strain-amplitude dependence of the opening angle *A* and cross angle *B* for the 10 wt % CNC suspension. The dashed line indicates γ = 10.

To further characterize the lobe geometry, the opening angle *A* and its complementary cross angle *B* were calculated from the peak azimuthal angles μ_45_, μ_135_, μ_225_, and μ_315_; the explicit definitions are provided in the Supporting Information. Figure 12(b) shows the strain-amplitude dependences of *A* and *B* for the 10 wt % CNC suspension. The lobe configuration observed at 10 wt % deviates from the ideal ±45° arrangement, indicating that the structure is not simply characterized by an increase in the anisotropy intensity but reflects selective orientation directions constrained by the elastic network and mesoscopic clusters. Immediately after the appearance of the four lobes, *A* ≈ 78° and *B* ≈ 102°, both angles remain nearly constant for γ ≲ 10. At higher strain amplitudes (γ ≳ 10), *A* gradually decreases while *B* correspondingly increases, demonstrating a continuous evolution of the four-lobe geometry under large deformation. This evolution is difficult to capture by only a qualitative observation but is clearly revealed by a quantitative annular-intensity analysis.

For the 8.5 wt % suspension, the formation of X-shaped scattering patterns is also confirmed, with *A* ≈ 73° and *B* ≈ 103°, values different from those observed for the 10 wt % system. However, no systematic change in the opening or cross angles was detected within the experimentally accessible strain-amplitude range, indicating that although X-shaped anisotropic SALS patterns appear, the continuous rearrangement of lobe geometry observed at high strain amplitude for the 10 wt % system is not sufficiently activated at 8.5 wt %. At concentrations of 7 wt % and lower, the four-lobe peaks could not be clearly identified within the experimentally accessible strain-amplitude range because of overlap with the background scattering and streak components. We therefore cannot exclude the possibility that clearer four-lobed patterns may appear at 7 wt % at higher strain amplitudes than those accessible here. Accordingly, quantitative analysis of the opening and cross angles was restricted to samples and strain-amplitude ranges in which all four peaks could be reproducibly identified within the same *q* range. A systematic comparison with analogous in situ Rheo-PI/Rheo-SALS measurements under steady shear was not performed in the present study. Therefore, the present interpretation of the anisotropic scattering evolution is restricted to the fixed-frequency LAOS protocol employed here.

### Comparative Relationship among Viscosity Crossover, Gelation, Local Optical Anisotropy, and Anisotropic SALS Signatures

The present results show that aqueous cellulose nanocrystal (CNC) suspensions exhibit distinguishable mechanical, optical, and scattering responses in the same system. Rather than appearing at a single concentration or strain-amplitude threshold, viscosity crossover, the sol–gel transition, mechanical yielding, the onset of detectable local optical anisotropy, and the appearance of anisotropic SALS signatures occur over different concentration and strain-amplitude ranges. Within the present fixed-frequency LAOS protocol, these observables are therefore treated as related but noncoincident signatures of deformation-induced organization across different length scales.

With respect to concentration, the empirical viscosity-crossover concentration *C*_cross_, obtained from the double power-law behavior of η_low_, marks the crossover in the concentration dependence of the operational low-rate viscosity, whereas the sol–gel transition concentration *C*_g_, defined by the Winter–Chambon criterion, corresponds to the emergence of an infinitely connected elastic network. Because *C*_cross_ and *C*_g_ do not coincide, the concentration at which viscosity begins to increase more steeply should be distinguished from the concentration at which elastic connectivity becomes mechanically detectable.

A similar distinction is observed with respect to strain amplitude under LAOS. The mechanical critical strain γ_c_, defined from the stress–strain curves, characterizes the transition from a solid-like elastic response to a plasto-viscoelastic response. Within the present protocol, γ_c_ is used as an operational mechanical yielding threshold, but it does not by itself specify when optical anisotropy or anisotropic SALS signatures first become detectable. The critical strains γ_PI1_ and γ_PI2_, defined by Rheo-PI, capture the onset and subsequent progression of optical anisotropy associated with local orientation. In contrast, γ_SALS1_ is defined operationally from the apparent contraction of initially circular or weakly distorted HV scattering patterns, whereas γ_SALS2_ is defined from the appearance of clearly identifiable X-shaped scattering patterns.

Accordingly, the mechanical, optical, and scattering thresholds are not treated here as interchangeable definitions of a single transition but as different response markers under the present experimental conditions. These quantities are summarized in Table 1 and their relationships are shown schematically in Figure 13.

**Figure 13:**
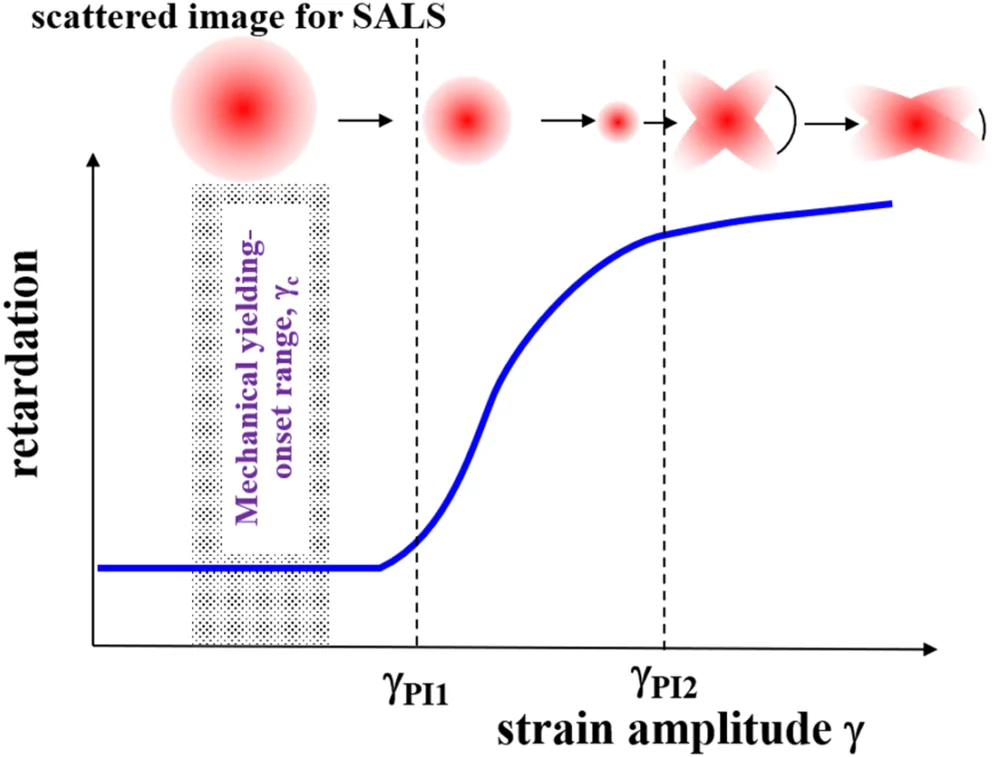
Schematic summary of viscosity crossover, sol–gel transition, mechanical yielding, local optical anisotropy, and anisotropic SALS signatures in aqueous CNC suspensions under fixed-frequency LAOS. The shaded region indicates the range of mechanical yielding-onset strains, γc, determined from the stress–strain curves under the same LAOS protocol.

**Table 1:** Operational Definitions and Measured Values or Ranges of the Reference Quantities Used in This Study^a^

quantity	operational definition	measured value or range	note
*C*_cross_	empirical intersection of two power-law regimes in η_low_(C)	≈2.6 wt %	operational viscosity-crossover concentration
*C*_g_	approximate sol–gel transition concentration from Winter–Chambon-type frequency dependence	≈4.5 wt %	approximate midpoint of the 4–5 wt % transition range
γ_c_	onset of nonlinear mechanical response from deviation of σ–γ curve from low-strain linearity	≈0.045–0.4	operational mechanical yielding-onset strain
γ_PI1_	first rapid increase in annularly averaged Δ_PI_	≈0.16–1.6	first Rheo-PI optical transition
γ_PI2_	strain where Δ_PI_ growth changes from rapid to gradual	≈1.0–16.0	second Rheo-PI optical transition
γ_SALS1_	apparent contraction of initially circular or weakly distorted HV SALS halo	≈0.29–1.0	operational SALS marker, not direct proof of correlation-length growth
γ_SALS2_	appearance of a clearly identifiable X-shaped HV SALS pattern	≈2.8–3.2	later SALS marker for directional mesoscopic anisotropy

a For concentration-dependent strain markers, the values are given as approximate measured ranges over the concentrations at which each marker was identified. The strain amplitudes are dimensionless nominal strain amplitudes under the present fixed-frequency LAOS protocol. Full concentration dependences are shown in Figures 6 and 9.

To facilitate comparison with the rheological response, the range of mechanical yielding-onset strains, γ_c_, determined from the stress–strain curves is also shown as a shaded region. At low strain amplitudes, the optical retardation remains nearly unchanged, and the SALS pattern remains circular without a noticeable change in size, indicating isotropic orientation and the absence of pronounced directional scattering signatures. As the strain amplitude approaches γ_PI1_, the retardation increases sharply, whereas the initially circular SALS pattern shows an apparent contraction. This apparent contraction is treated as an operational SALS marker rather than direct proof of correlation-length growth. At γ_PI2_, the SALS pattern changes from circular or weakly distorted to X-shaped, indicating that larger-scale directional scattering anisotropy becomes detectable only after local optical anisotropy has already developed. At still higher strain amplitudes, the lobe angles change continuously, reflecting further evolution of the selected orientational correlations. Within the present fixed-frequency LAOS protocol, Rheo-PI therefore reports the earlier detectability of local optical anisotropy, whereas Rheo-SALS reports the later appearance and evolution of larger-scale directional scattering signatures.

Overall, the combined rheological, optical, and SALS measurements provide a comparative multiscale framework for distinguishing viscosity crossover, gelation, mechanical yielding, local optical anisotropy, and anisotropic light scattering signatures in aqueous CNC suspensions. Rather than treating these observables as a single coincident transition, the present results show that they become detectable over different concentration and strain-amplitude ranges under the present experimental conditions. This distinction is important for interpreting the deformation-induced organization in surface-charged rod-like colloidal gels.

## Conclusions

In this study, we performed a hierarchical investigation of gelation and nonlinear responses in aqueous suspensions of sulfated cellulose nanocrystals (CNCs) by combining linear and nonlinear rheology with rheo-polarized imaging (Rheo-PI) and rheo-small-angle light scattering (Rheo-SALS). Analysis based on the operational low-rate viscosity and the Winter–Chambon criterion revealed that the concentration marking the viscosity crossover is distinct from that at which an infinitely connected elastic network is formed. Accordingly, an empirical viscosity-crossover concentration, *C*_cross_, should be distinguished from the sol–gel transition concentration, *C*_g_, in the present aqueous CNC suspensions. Furthermore, we demonstrated that the mechanically, optically, and structurally defined critical strains—identified from stress–strain curves, Rheo-PI, and Rheo-SALS, respectively—do not coincide.

Rheo-polarized imaging (Rheo-PI) revealed that optical anisotropy arising from local CNC orientation can already be detected at relatively low concentrations and low strain amplitudes, including conditions where elastic connectivity is not yet established and mechanical yield has not yet occurred. In contrast, Rheo-small-angle light scattering (Rheo-SALS) showed that clearly identifiable X-shaped anisotropic scattering patterns appear only under larger deformation, after the earlier Rheo-PI optical response and the apparent contraction of the HV scattering halo. Under the present fixed-frequency LAOS protocol, Rheo-PI therefore serves as an earlier optical readout of local anisotropy, whereas Rheo-SALS reports the later appearance of larger-scale directional scattering signatures.

These observations indicate that the local optical anisotropy, anisotropic SALS signatures, and mechanical network response become detectable over distinct concentration and strain-amplitude ranges. Rather than treating these observables as a single coincident transition, the present results provide a comparative multiscale framework for distinguishing viscosity crossover, gelation, mechanical yielding, local optical anisotropy, and anisotropic light scattering signatures in aqueous CNC suspensions.

The present results indicate that elastic network formation and the appearance of deformation-induced anisotropic mesoscopic scattering signatures in CNC suspensions do not necessarily coincide and become detectable under distinct concentration and strain-amplitude conditions. More broadly, the comparative framework presented here may be useful for understanding nonlinear responses and structural evolution in rod-like colloidal gels with orientational degrees of freedom, including but not limited to nanocellulose systems.

## Supplementary Material







## Data Availability

All data generated or analyzed during this study are included in this published article and its Supporting Information files.

## Notes

Rheo-PI movie of a 5 wt % CNC suspension under LAOS (MP4)
Simultaneous rheology and Rheo-SALS movie of a 10 wt % CNC suspension under LAOS (MP4)
Experimental details and supporting rheological, Rheo-PI, and Rheo-SALS data, including exposure time and gain settings, reproducibility tests, stress–strain analysis, VFT-fitting sensitivity analysis, alternative mechanical-transition criteria, phase-unwrapping procedure, and annular-intensity analysis (PDF)
