# Supplementary material for: Multiscale Rheo-Optical and Scattering Signatures of Deformation-Induced Anisotropy in Aqueous Cellulose Nanocrystal Suspensions under Large-Amplitude Oscillatory Shear
Source: Langmuir. 2026 Sep 25;42(40):29867–80. doi: 10.1021/acs.langmuir.6c03702 (PMC13674231; doi:10.1021/acs.langmuir.6c03702)
Supplement: Supplementary file 1 [file la6c03702_si_003.pdf]

Supporting Information

Multiscale Rheo-Optical and Scattering Signatures of Deformation-Induced Anisotropy in Aqueous Cellulose Nanocrystal Suspensions under Large-Amplitude Oscillatory Shear

*Yoshifumi Yamagata[1, 2], Ainaa Amirah Marzuki[3], Saki Otobe[1], Moe Araida[1], Taisuke Sato[4]

Number of pages: 7

Number of figures: 6

Number of schemes: 0

Number of tables: 3

Table of Contents

Table S1. Exposure time and gain for obtaining appropriate scattering intensity.

Table S2. Sensitivity of VFT-type fitting to the upper-bound constraint on C_0.

Table S3. Alternative mechanical transition strains determined from LAOS data.

Figure S1. (a) Time-sweep measurements of G' and G" for a re-prepared 5 wt% CNC suspension at ω = 0.1 rad s^{-1} and γ = 1% and 0.5%. After a short initial transient, both moduli reach a steady plateau. (b) Reproducibility of frequency-sweep measurements at γ = 1% for three independently prepared samples. Data below ω = 0.1 rad s^{-1} are not shown due to the instrument torque resolution limit.

Figure S2. Stress-strain curve obtained from LAOS measurement. The red arrow indicates the point where the slope deviates from 1. A broad later bend is also observed near $\gamma \approx 1$ for the higher-concentration samples and is discussed in the main text as a later large-deformation mechanical response.

Figure S3. Changes in elastic moduli and polarized imaging patterns of 5 wt% CNC suspension under LAOS measurement.

Figure S4. Schematic illustration of the phase-unwrapping procedure used to reconstruct the true optical retardation.

Figure S5. Changes in elastic moduli and scattering patterns of 10 wt% CNC suspension under LAOS measurement.

Figure S6. Annular intensity and definition of azimuth angle (10 wt% CNC at strain of 100).

Table S1. Exposure time and gain for obtaining appropriate scattering intensity.

CNC concentration (wt%)	Exposure time (ms)	Gain
6	10	3
7	10	3
8.5	10	3
10	10	1.5

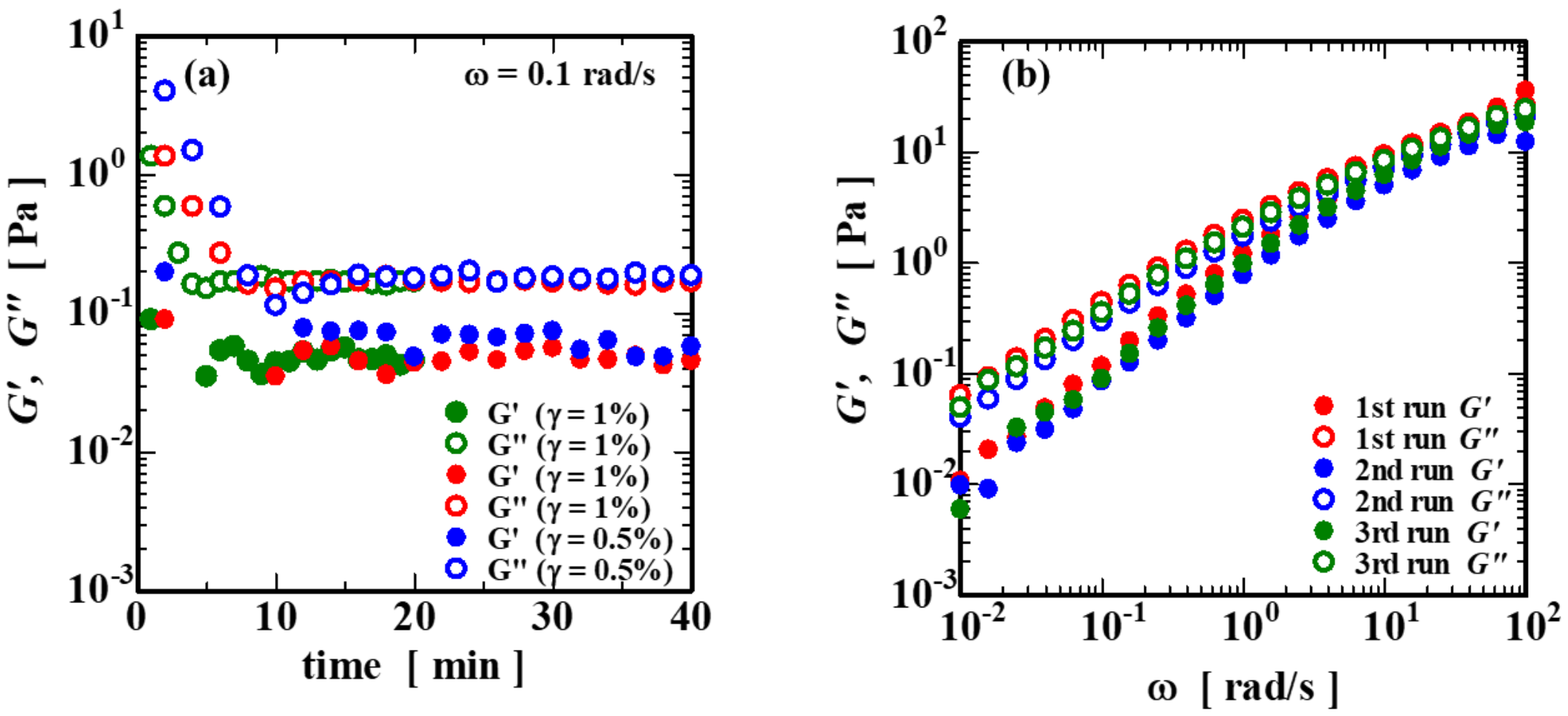

Fig. S1 (a) Time-sweep measurements of G' and G" for a re-prepared 5 wt% CNC suspension at ω = 0.1 rad s^{-1} and γ = 1% and 0.5%. After a short initial transient, both moduli reach a steady plateau. (b) Reproducibility of frequency-sweep measurements at γ = 1% for three independently prepared samples. Data below ω = 0.1 rad s^{-1} are not shown due to the instrument torque resolution limit.

To assess possible structural evolution at low frequency and the reproducibility of the linear viscoelastic measurements, a 5 wt% CNC suspension was re-prepared and independently tested. Owing to slight variations in sample preparation, the absolute values of the moduli were somewhat lower than those reported in the main manuscript; however, this does not affect the evaluation of time dependence and reproducibility.

(a) In time-sweep measurements at ω = 0.1 rad s^{-1}, G' and G" were monitored at γ = 1% for 20 and 40 min, and additionally at γ = 0.5% for 40 min. In all cases, neither G' nor G" exhibited any systematic drift or monotonic evolution over the measurement period. After a short initial transient regime following sample loading, both moduli reached a quasi-steady plateau, indicating the absence of appreciable time-dependent structural evolution under these conditions. Each measurement was repeated three times using independently prepared samples, and consistent results were obtained.

(b) Frequency sweeps at γ = 1% were also repeated three times, and the resulting datasets nearly overlapped across the accessible frequency range, demonstrating good reproducibility. At ω < 0.1 rad s^{-1}, the

measured torque fell below the instrument resolution limit; therefore, data in this range are not shown and were excluded from further analysis.

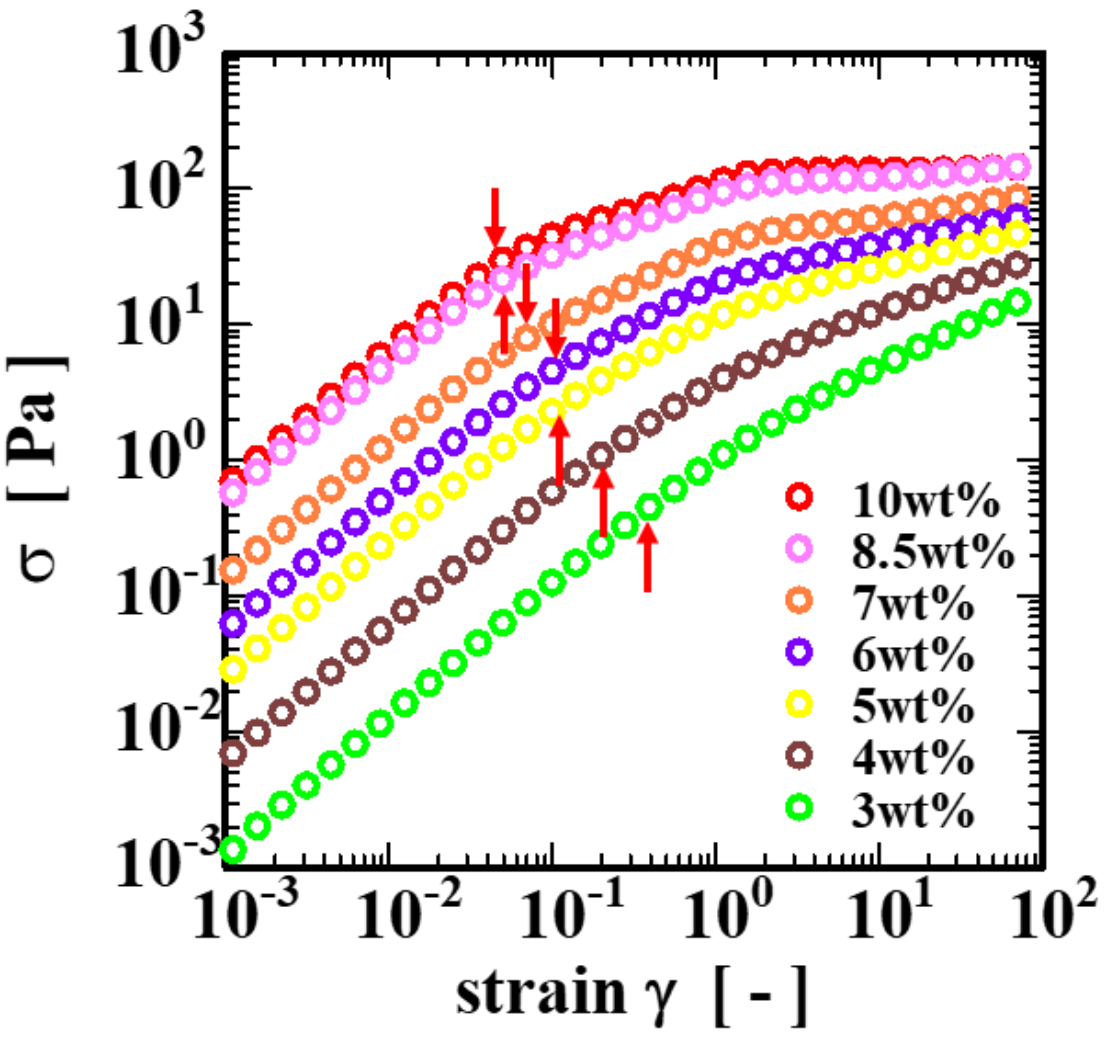

Fig. S2. Stress-strain curve obtained from LAOS measurement.

The red arrow indicates the point where the slope deviates from 1. A broad later bend is also observed near $\gamma \approx 1$ for the higher-concentration samples and is discussed in the main text as a later large-deformation mechanical response.

Table S2. Sensitivity of VFT-type fitting to the upper-bound constraint on C_0.

Constraint on C_0	Optimized C_0 / wt %	SSE	R^2 for $\ln\eta_{app}$
$C_0 \leq 12$ wt %	12.000	22.424	0.679
$C_0 \leq 15$ wt %	15.000	12.501	0.821
$C_0 \leq 20$ wt %	20.000	7.722	0.889
$C_0 \leq 50$ wt %	50.000	3.674	0.947
$C_0 \leq 100$ wt %	92.866	2.978	0.957
No upper bound on C_0	190.232	2.634	0.962

The VFT-type fitting was performed using the apparent viscosity at 0.01 s^{-1} in the concentration range of 3–10 wt %. The results show that the optimized C0 depends strongly on the imposed upper-bound constraint and lies far outside the measured concentration range when the constraint is relaxed.

Table S3. Alternative mechanical transition strains determined from LAOS data.

CNC concentration wt %	Strain at maximum G’γ	Strain at maximum G”	G’=G” crossover strain
3	1.42	0.0318	n.o.
4	1.42	0.0179	n.o.
5	1.27	0.00357	0.100
6	1.01	0.00113	0.159
7	1.01	0.0798	0.283
8.5	1.01	0.101	0.318
10	1.01	0.127	0.400

Note. All values are strain amplitudes under the fixed-frequency LAOS protocol.

The columns labeled “Strain at maximum G’γ” and “Strain at maximum G”” denote the strain amplitudes at which the elastic stress amplitude G’γ and the loss modulus G”, respectively, reach their maximum values. The G’ = G” crossover strain is reported only when the crossover was observed within the measured strain-amplitude range.

n.o. indicates not observed within the measured strain-amplitude range.

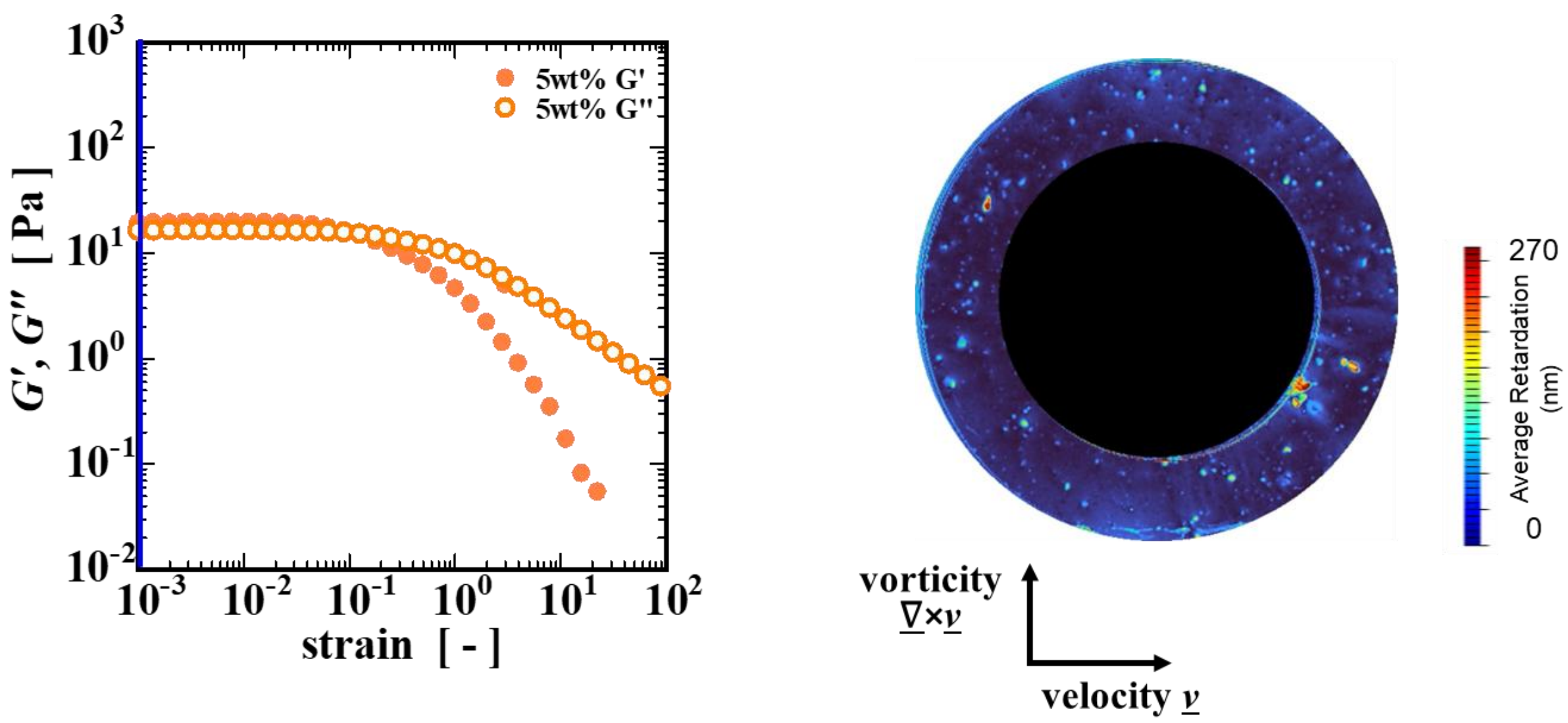

Fig. S3. Changes in elastic moduli and polarized imaging patterns of 5 wt% CNC suspension under LAOS measurement.

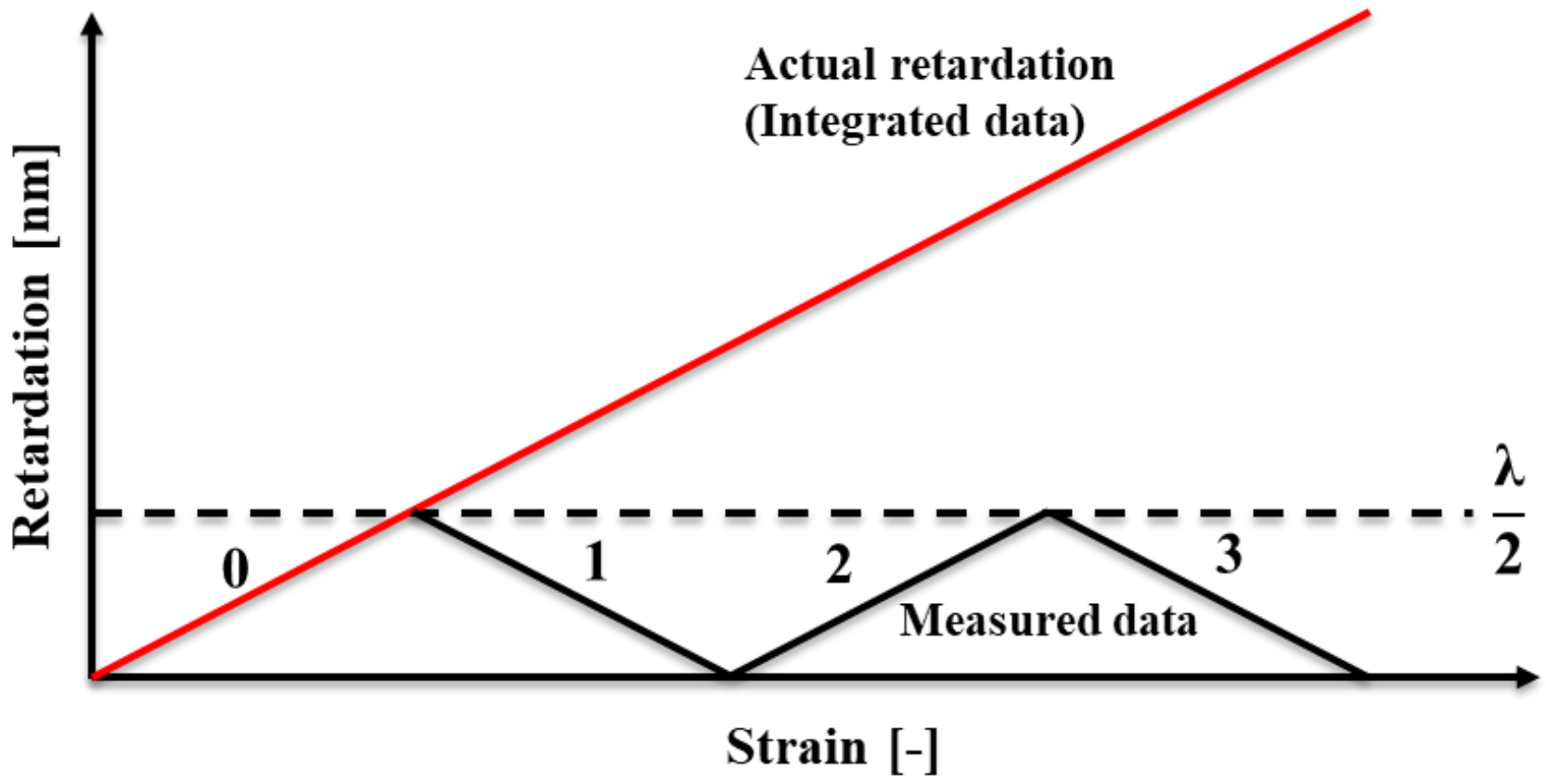

Fig. S4. Schematic illustration of the phase-unwrapping procedure used to reconstruct the true optical retardation.

The true optical retardation $\Delta_{\text{integrated}}$ was reconstructed from the measured retardation Δ_{measured} using the following relations:

$$\Delta_{\text{integrated}} = n \times \frac{\lambda_{\text{PI}}}{2} + \Delta_{\text{measured}} \qquad \text{(S1)}$$

(n = 0, 2, 4, · · · even numbers)

$$\Delta_{\text{integrated}} = n \times \frac{\lambda_{\text{PI}}}{2} + \left(\frac{\lambda_{\text{PI}}}{2} - \Delta_{\text{measured}}\right) \qquad \text{(S2)}$$

(n = 1, 3, 5, · · · odd numbers)

Figure S4 illustrates the phase-unwrapping procedure used to reconstruct the true optical retardation when apparent inversion occurs in the present polarization-imaging measurements. Because the measured retardation is periodic with respect to the true optical retardation, the apparent signal folds once it exceeds the principal measurable range of the present optical configuration. The true retardation is reconstructed by adding integer multiples of $\lambda_{\text{PI}}/2$ to the measured retardation, according to Eqs. (S1) and (S2), following standard treatments of phase wrapping in polarization-based retardation measurements.[28–30]

[28] Kemp, J.C. Piezo-optical birefringence modulators: New use for a long-known effect, J. Opt. Soc. Am. 1969, 59, 950-954.

[29] Hinds Instruments, Light intensity modulation using a PEM, Application Note. https://www.hindsinstruments.com/wp-content/uploads/Light-Intensity-Modulation-Using-a-PEM-Application-Note.pdf. (accessed January 22, 2026)

[30] Aben, A.; Guillemet, C. Photoelasticity of Glass, Springer, 1993

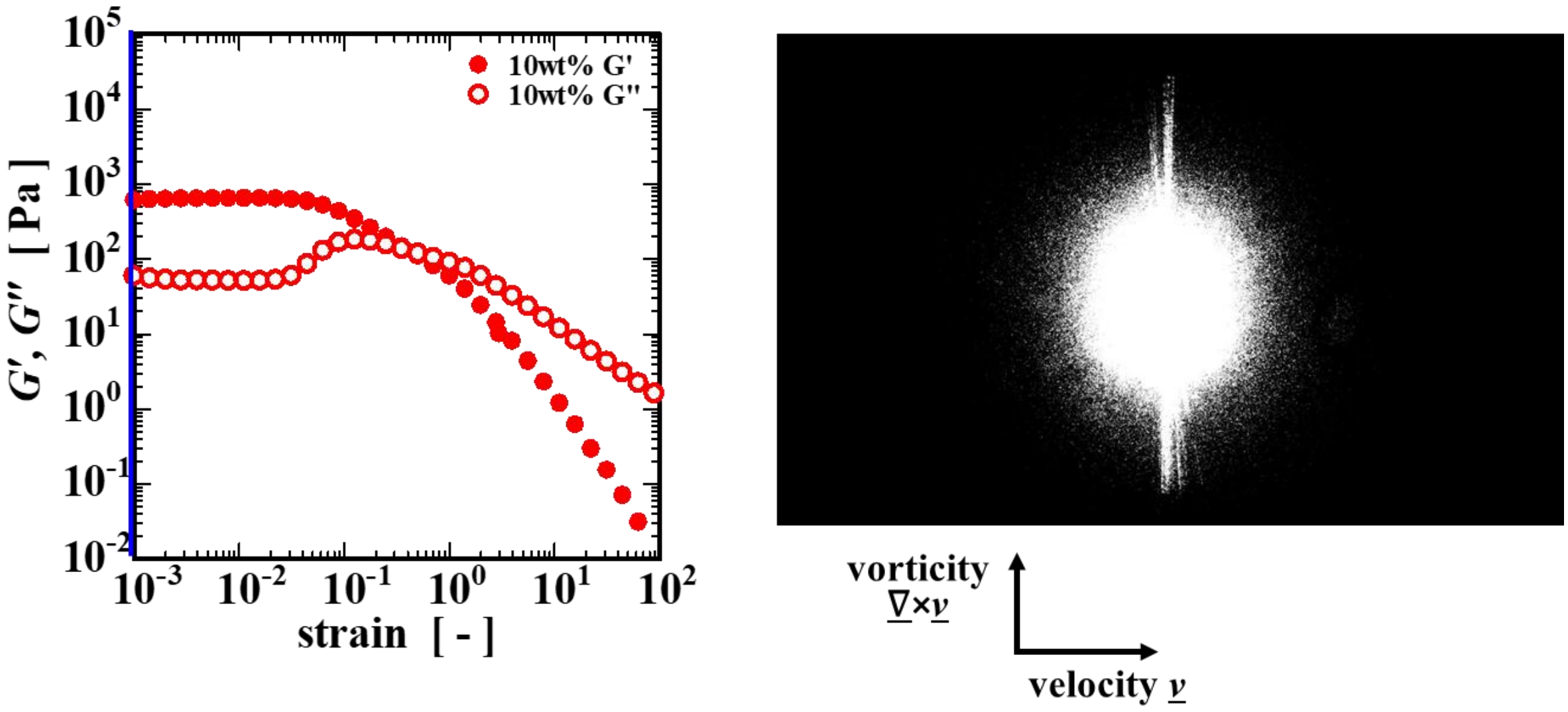

Fig. S5. Changes in elastic moduli and scattering patterns of 10 wt% CNC suspension under LAOS measurement.

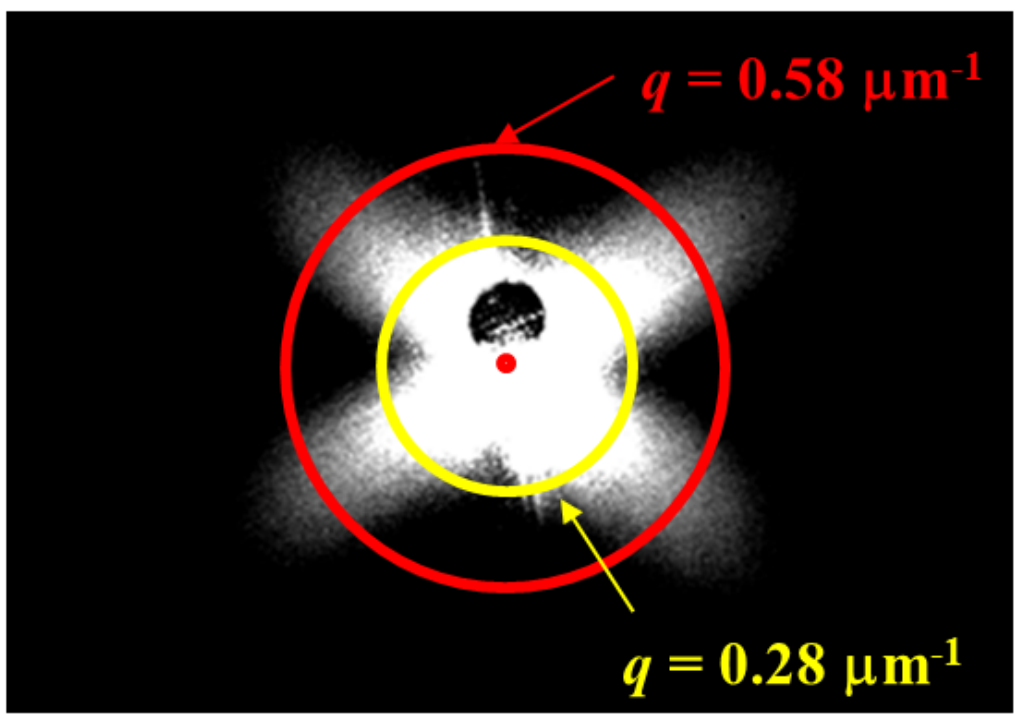

(a) Range of scattering vectors used for calculating annular intensity.

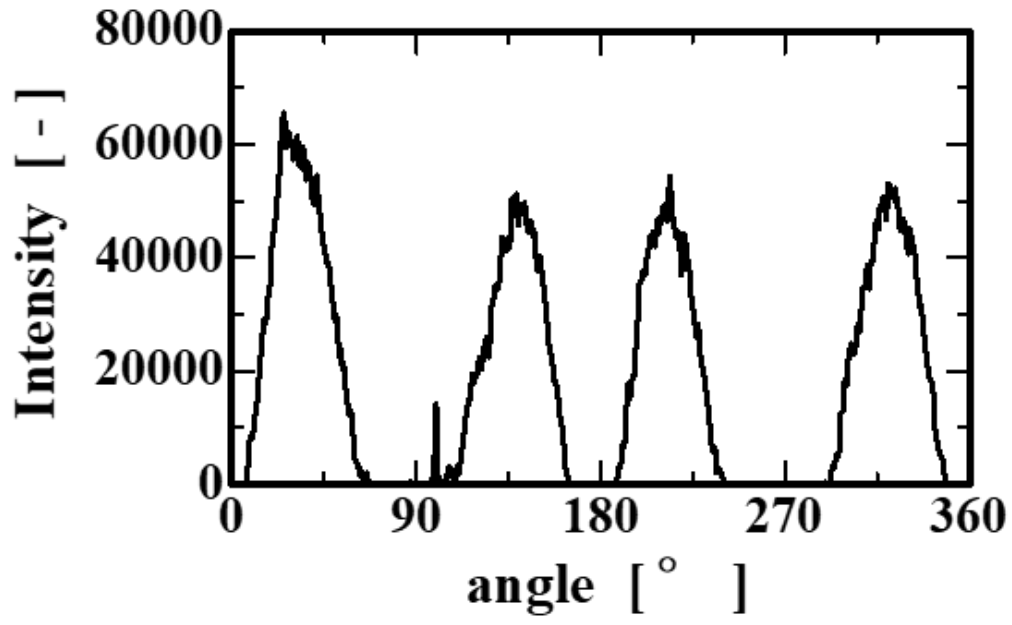

(b) Annular intensity at $q = 0.58$ μm^{-1}.

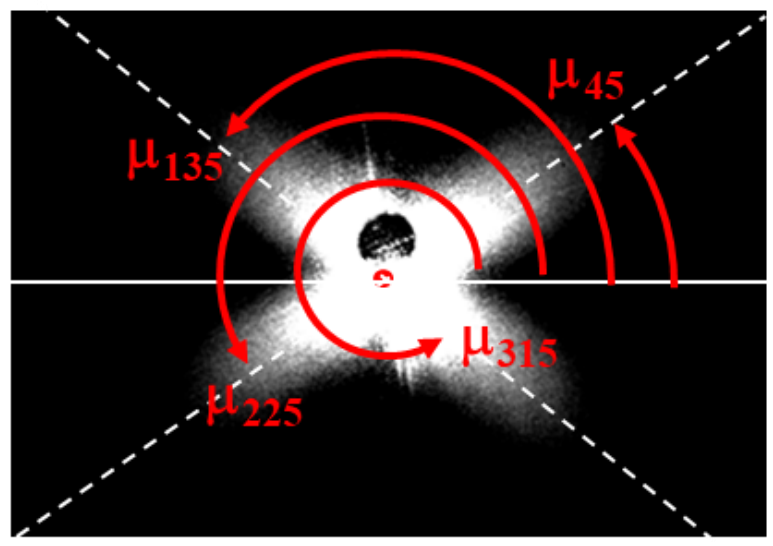

(c) Definition of azimuth angle.

Fig. S6. Annular intensity and definition of azimuth angle (10 wt% CNC at strain of 100).

The opening angle A and complementary cross angle B were calculated from the peak azimuthal angles μ_{45}, μ_{135}, μ_{225}, and μ_{315} as follows [31]:

$$A = \frac{[\{\mu_{225}-\mu_{135}\} + \{\mu_{45}+(360-\mu_{315})\}]}{2}$$

$$B = \frac{\{(\mu_{135}-\mu_{45})+(\mu_{315}-\mu_{225})\}}{2}$$

[31] Moses, E.; Kume, T.; Hashimoto, T. Shear Microscopy of the "Butterfly Pattern" in Polymer Mixtures. Phys. Rev. Lett. 1994, 72 (13), 2037–2040.
